# A privacy and security analysis of early-deployed COVID-19 contact tracing Android apps

**DOI:** 10.1007/s10664-020-09934-4

**Published:** 2021-03-19

**Authors:** Majid Hatamian, Samuel Wairimu, Nurul Momen, Lothar Fritsch

**Affiliations:** 1 Department of Computer and Information Sciences, Northumbria University, Newcastle upon Tyne, UK; 2 Department of Mathematics and Computer Science, Karlstad University, Karlstad, Sweden; 3 Blekinge Institute of Technology, Karlskrona, Sweden

**Keywords:** COVID-19, Contact tracing app, Privacy, Security, Vulnerability, GDPR, Pandemic

## Abstract

As this article is being drafted, the SARS-CoV-2/COVID-19 pandemic is causing harm and disruption across the world. Many countries aimed at supporting their contact tracers with the use of digital contact tracing apps in order to manage and control the spread of the virus. Their idea is the automatic registration of meetings between smartphone owners for the quicker processing of infection chains. To date, there are many contact tracing apps that have already been launched and used in 2020. There has been a lot of speculations about the privacy and security aspects of these apps and their potential violation of data protection principles. Therefore, the developers of these apps are constantly criticized because of undermining users’ privacy, neglecting essential privacy and security requirements, and developing apps under time pressure without considering privacy- and security-by-design. In this study, we analyze the privacy and security performance of 28 contact tracing apps available on Android platform from various perspectives, including their code’s privileges, promises made in their privacy policies, and static and dynamic performances. Our methodology is based on the collection of various types of data concerning these 28 apps, namely permission requests, privacy policy texts, run-time resource accesses, and existing security vulnerabilities. Based on the analysis of these data, we quantify and assess the impact of these apps on users’ privacy. We aimed at providing a quick and systematic inspection of the earliest contact tracing apps that have been deployed on multiple continents. Our findings have revealed that the developers of these apps need to take more cautionary steps to ensure code quality and to address security and privacy vulnerabilities. They should more consciously follow legal requirements with respect to apps’ permission declarations, privacy principles, and privacy policy contents.

## Introduction

As this paper is being written, the COVID-19 pandemic has spread across the world (Trackcorona 2020). To manage and control the pandemic, countries and regions are taking different approaches. Enacting partial to full lockdown (with an exception of countries like Sweden), mandating safe physical-distancing measures, face mask wearing for general public, measures for closing/reopening schools and universities, encouraging remote working, border control, using manual and digital contact tracing, and using hygiene measures are among the widely adopted strategies to the pandemic (Han et al. 2020).

Many countries started the the introduction of contact tracing apps to manage and control the spread of the virus (EDPB 2020a). Contact tracing apps should complement and support the manual contact tracing as such a technology may not be able to penetrate in some populations (e.g. children or elderly). Further, in some countries people may not have access to smartphones and mobile devices to install such apps. Accordingly, manual contact tracing remains the main method of contact tracing (EDPB 2020a; Ferretti et al. 2020).

To date, there are many contact tracing apps^1^ and many of them hastily designed, developed, and produced (Covid-19 apps 2020).

These apps while essentially beneficial - in terms of generating a memory of proximity identifiers and urgently alerting users if they come into contact with a COVID-19 positive case (Ferretti et al. 2020) - present substantial security and privacy challenges. In this article, we analyze the privacy and security aspects of COVID-19 contact tracing apps (a set of 28 apps available on Android platform) by applying a multilateral analysis method for Android apps introduced by Hatamian et al. (2019). Through this method, we analyze the permissions declared in the Android manifest files. In particular, we focus on the dangerous permissions. We analyze the privacy policies of these apps. We monitor app behavior by logging in each resource access event during run-time; here we fundamentally focus on dangerous permissions even though the monitoring app documents other types of resource access events. Lastly, to complement the privacy analysis we perform a security analysis of each app’s program code in order to detect possible vulnerabilities. The period of our analysis was May-June 2020, we collected data for accessible apps for this study, archived their documentation, installed them on test phones in our labs, and started analyzing their behavior.

### Research questions.

Researchers have pointed out the privacy and security challenges of COVID-19 apps (Dar et al. 2020; Raskar et al. 2020). However, in this case, our motivation is driven by several fundamental questions: What privacy sensitive data do the COVID-19 apps aim to extract from the users? Does the apps’ behavior match what has been indicated in their privacy policies? Are there vulnerabilities that would undermine the information security and privacy protection goals? To which extent the studied apps are compliant with legal requirements (especially the EU General Data Protection Regulation (GDPR) as this study has been conducted in EU)? To address these questions, we develop a method that combines four metrics: *(i)* apps’ data access potential from their permission requests, *(ii)* dynamic app-behavior analysis by monitoring their run-time resource accesses patterns, *(iii)* coverage of data protection principles by their corresponding privacy policy texts, and *(iv)* static app-code analysis to document existing security vulnerabilities. The collected data is used as a basis for a multi-perspective privacy and security analysis of COVID-19 contact tracing apps, enabling an understanding of privacy and security related quality indicators for rapidly deployed smartphone apps within the context of a health pandemic. It should be noted that the privacy and security analysis scope of this paper is focused on Android ecosystem and the reason is threefold: (1) the open-source nature of Android and its flexibility in modifying the core components of the operating system to monitor apps’ behavior along with the existence of a wide range of static and dynamic analysis tools (2) compared to other mobile operating systems, Android is globally used by many users, thanks to its domination of the market share (Mobile operating system market share worldwide 2020; Statista 2020; IDC 2020).Furthermore, it is among the operating systems with the most detected vulnerabilities (Android is the most vulnerable operating system 2019; DigitalInformationWorld 2019), making it an attractive platform for adversaries to conduct malicious activities, and (3) during the data collection phase (see Section 3.1), we noticed that all studied contact tracing apps are available on Android platform (28 out of 28 apps), while some of them were not available on iOS platform (6 out of 28 apps).

### Structure of the paper.

The rest of this paper is organized as follows: Section 2 provides background information regarding the available COVID-19 contact tracing apps including the technologies they rely on. It also provides some insights into compatability of these apps with privacy regulation. While Section 3 elaborates on the steps that were taken into consideration to design our study, Section 4 details our multi-perspective privacy and security analysis. Further, based on the results obtained from the multi-perspective analysis, Section 5 provides a holistic view through proposing and conducting an impact assessment to compare the privacy and security performance of contact tracing apps. Section 6 discusses the main key insights, examines the compliance of COVID-19 contact tracing apps with respect to fundamental privacy and security requirements, and provides several calls for actions to revamp the identified issues. This paper is then concluded in Section 7.

## COVID-19 contact tracing apps

Contact tracing is one of the methods used to contain a medical epidemic. By tracing humans exposed to an infected person, the spread of infections can be reduced if those potentially infected people can be isolated from the remaining population. In addition, contact tracing helps in tracking the areas that are exposed to an infection (EDPB 2020a). In what follows, we briefly discuss the existing technologies used in contact tracing apps along with the compatibility of such apps with privacy regulation.

### Existing technologies

During the emergence of the 2019-2020 pandemic disease COVID-19 caused by the SARS-CoV-2 virus which spread at high speed, governments turned towards digital tracing of their populations with the help of smartphone apps. The principal idea is that large parts of a population carry phones with them, thus phones could be used as sensors for both recording encounters between people as well as for registration of their whereabouts. Smartphone location tracking is not a new concept (Fritsch 2008a). Mobile phones (including both smart and non-smart ones) send and receive low power radio signals. The signals are sent to and received from antennas that are attached to radio transmitters and receivers, usually referred to as base stations. The base stations are linked to the rest of the mobile and fixed phone cellular networks and pass the signal/call on into those networks. Therefore, as part of services, mobile phones are tracked as they move across different cellular networks (Al-Saffar et al. 2015). However, the sensing capabilities of phones have exceeded that of phone networks both in precision and in the quality of information: GPS delivers more precise location data, while scanning each other’s radio interfaces provides proximity information about close encounters to other phones. The latter is used in Bluetooth contact tracing, where the short-range wireless Bluetooth Low Energy (BLE) technology is used to send and detect beacon signals in order to register phone encounters. Moreover, it is an established practice in marketing and customer intelligence gathering, and is widely used e.g. in digital media and radio distribution in order to identify stations, products or billboards (Lashgari 2018; Rocamora 2017). Analyzing and processing of sensor-generated data (through sensors like accelerometer, gyroscope, etc.) and ultrasonic signals through embedded sensors (microphone) sent by other phones are other technique for digital contact tracing (eHealthNetwork 2020). In the case of ultrasonic signals processing, one can expect more reliable accuracy than BLE and GPS (Luo et al. 2018) as they both measure signals through walls and floors which could produce false positives indoors. However, this is not the case for ultrasonic signals as they will not travel through walls, floors, etc. Nevertheless, privacy concerns are not avoidable (ultra-privacy 2020)because of its reliance on the microphone (there is no guarantee that only ultrasonic signals are collected).

In Table 1 the list of contact tracing apps analyzed and studied is available. This table also details the technologies used in each app. As can be seen, each GPS and BLE has accounted for 60% (17 apps) of all studied apps. This percentage is 10% (3 apps) for sensor-based technologies (e.g. gyroscope, microphone, etc.). Our analysis shows that the reviewed apps require a mixed variety of personal data to function, including precise location information, data generated by sensors, phone number, gender, age, devices’ unique IDs, etc. We argue that depending on the technologies used, there might be a trade-off between privacy and functionality. According to existing guidelines (EDPB-letter 2020), no usage of GPS and use of decentralized storage is well-aligned with best design practices as apps will not be able to track and monitor users’ precise movement patterns and store these data on centralized storage. Additionally, the capability of devices to determine social distancing varies depending on technologies used. For instance, when it comes to indoors positioning, ultrasonic-based technologies are more reliable in terms of distance accuracy. On top of that, potential interference and spatial blockage between different devices is another challenge that needs further research and development (Omar Al Kalaa et al. 2016; Tshiluna et al. 2016).

**Table 1:** Collected data set: apps, country of origin, technology, functionality

#	Country	App	Technology			How it functions
			GPS	BLE	Sensors	
1	Australia	COVIDSafe		X		Based on BLE, it records anyone a user gets close to who also has the app. The two apps exchange IDs. If someone is tested positive, they then get a unique code from a health official via SMS to use in the app to consent to upload the list of anonymised IDs for the past 21 days of contact for contact tracing. It uses signal strength and other data then to work out who needs to be contacted.
2	Austria	Stopp Corona		X		Determines and compares the sent/ received signal strengths between users. If similar, it draws the conclusion that the two phones are in close proximity. The app processes timestamps, app-ID, user-ID, OS version, device model, and Wifi access point MAC addresses.
3	Brazil	Coronavirus-SUS	X			Logs users’ location data and daily reporting of symptoms together with other personal data (age, name, medical history, etc.) and traces the spread of the virus.
4	Columbia	CoronApp	X			Logs users’ location data and daily reporting of symptoms together with other personal data (age, name, medical history, etc.) and traces the spread of the virus.
5	Czech Republic	eRouska		X		Uses BLE to log phones that the user has had close contacts with. Once someone is tested positive, it notifies other users to test and isolate.
6	Germany	Ito		X		Uses BLE to log phones that the user has had close contacts with. Upon a positive test, contacted users receive a code from the health department to enter in the app. The code is then be uploaded together with the verification of the positive test.
7	Hungary	VirusRadar		X		Uses BLE to communicate with other users and exchanges encrypted data about the distance of surrounding devices if they have been at a dangerous distance. If users become infected, they can share their data. The data forwarded to the authorities can be used to trace contacts patients had interacted with within a 2 meter distance for at least 20 minutes in the last 14 days.
8	Georgia	Stop Covid	X	X	X	Based on collecting location and proximity data, sensor data (gyroscope, acceleration, magnetometer), activity data provided by the operating system, and user-ID, it logs interactions that span more than 15 minutes and take place within two meters. If a user tests positive, he can inform the app, and can provide information pertaining to contacts in the last five days. All other users which have been in contact with him in the last five days will be notified by the app.
9	Iceland	Ranking C-19	X			Tracks users’ GPS data to compile a record of where they have been to look at whether those with a positive diagnosis are potentially spreading the disease.
10	India	Mahakavach	X			Collects location history, name, email, phone number, age, gender, and medical records and history to trace the geographical spots a user has been to in the last 14-20 days, and checks how many other people may have come in contact with and thus, possibly transmitted the virus to. It collects these data for other purposes than contact tracing (e.g. to generate reports, heat maps and other statistical visualizations).
11	India	COVA Punjab	X			Collects personal data such as demographics, IMEI/IMSI number, device ID, and movement patterns to trace the geographical spots a user has been to, and checks how many other people may have come in contact with and thus, possibly transmitted the virus to. If the user delets the app, he will still continue to be a registered user of the app and receive promotions /newsletters/ notifications.
12	India	Aarogya Setu	X	X		Works based on access to proximity (via BLE) and GPS information to alert people when they come in contact with someone who has tested positive.
13	Israel	Hamagen	X			Works based on GPS information and correlates location history to alert people when they come in contact with someone who has tested positive, including the exact time and location.
14	Italy	SM-Covid19	X	X		Uses BLE and GPS to monitor the number of contacts, duration of time with contacts, and distance between contacts.
15	Italy	diAry	X	X	X	Collects GPS, Wi-Fi, bluetooth, gyroscopes, oscillators, accelerometers and magnetometers data. Detects the position and the movements of the user. It calculates daily statistics of the time passed in each place or of each movement, recognizing if the movements are done by foot, bicycle or motorized vehicles. These data can be uploaded to a central database.
16	Jordan	Aman	X			Collects user location data to examine and compare movements of users in parallel to those of virus carriers already identified. Should a locational overlap occur between users and virus carriers later identified as patients, it alerts its users about a possible exposure to the virus and provides instructions about home isolation and contacting authorities. Once a user tested positive, it retraces the user’s movements and provides information such as dates, times, and places to notify other users who happened to be in the vicinity of the diagnosed patient.
17	Malaysia	Gerak	X			Requires personal details, name, address and email. Users also have to give permission to track their location at all times via the phone’s GPS.
18	Malaysia	MyTrace		X		Collects proximity data whenever the app detects another device with the same app installed. When a user is tested positive, it uploads the data from the user’s smartphone to a centralized database.
19	North Macedonia	StopKorona!		X		Uses BLE to exchange anonymized data with every other nearby users, measuring their mutual distance. It uses received signal strength indication values to measure signal strengths between telephones. These calibrated values are used to estimate approximate distance between users, whereas the duration of such connection is registered by the mobile app itself.
20	Norway	Smittestopp	X	X		Collects proximity (via BLE) and location data to detect other nearby phones with the app installed. Anyone defined as a close contact in the days prior to the diagnosis will receive an SMS.
21	Russia	Contact Tracer	X	X		Accesses location data through GPS, Bluetooth, and Wi-Fi signals to check users had been in close contact with individuals infected by the virus. It will identify those who contacted the person within 14 days.
22	Singapore	Trace Together		X		Collects phone number and user-ID and and exchanges Bluetooth signals between phones to detect other participating users in close proximity to notify users in case a positive case is detected.
23	South Africa	Covi-ID	X			Collects location, name, date of birth, gender, email, physical address, and phone numbers. Users are asked to enter their COVID-19 status. They are then assigned a QR code on their smartphones. When a user goes somewhere (e.g. to work), the QR code is scanned and he gets a so-called geolocation receipt that details where and when the user has been at a certain time.
24	UK	COVID Symptom Study	X			Collects body temperature, height and weight, gender, age, location, name, email phone number, IP address, and device ID and to measure how quickly the virus is spreading in different areas and identify areas and users at high risk.
25	US	COVID Safe		X		Collects proximity data and user-IDs. Users get notified if someone who was near them within the last two weeks has come down with symptoms of COVID-19.
26	US	PrivateKit	X			Collects location data and user-IDs, keeping a time-stamped log every five minutes to notify users in case a positive case is detected.
27	US	NOVID		X	X	Based on BLE and ultrasonic signals, it logs users proximity information when he spends some time physically close to someone else who has the the app. If a user tells the app if he has tested positive, people that have come into contact with that user recently will receive a notification.
28	Global	Coalition		X		Proximity data including the amount of exposure period and the length of time and user-IDs are collected. If a user is tested positive, all phones that have been in his proximity are informed.

### Compatibility with privacy regulation

The fast spread of SARS-CoV-2 produced many proposals and implementations of contact tracing apps. Some countries imposed tracking apps on their populations that implemented a varying degree of government force on the bearers, ranging from self-reporting duties through biometric surveillance up to publishing infected citizen’s movements on public web pages. For instance, the Indian authorities announced that the use of Aarogya Setu app is mandatory for federal government employees, food delivery workers, and some other service providers. Moreover, to access public transport and airports one needs to have it installed (IndiaMandatory 2020). In a similar scenario, Singapore contact tracing app (TraceTogether) became mandatory for migrant workers (Singapore 2020). Not quite irrelevant to this, a university in the US has shown an interest in mandating students to install a tracking app, otherwise they will face disciplinary proceedings and sanctions (US-University 2020).

In the European Union (EU), compatibility with privacy regulation and proportionality of measures were quickly pointed out by the European Commission when contact tracing started being discussed (EDPB 2020b). For instance, on 8th April 2020 the European Commission adopted a recommendation (COVID-europe 2020) towards a common Union toolbox for the use of technology and data to combat and exit from the COVID-19 crisis, in particular concerning mobile apps and the use of anonymized mobility data to develop a common European approach for the use of apps at an EU level. Followed by this, the European Data Protection Board published a public letter (EDPB-letter 2020) where it was stated that “contact tracing apps do not require location tracking of individual users. Their goal is not to follow the movements of individuals or to enforce prescriptions. The main function of such apps is to discover events (contacts with positive persons), which are only likely and for the majority of users may not even happen, especially in the de-escalation phase. Collecting an individual’s movements in the context of contact tracing apps would violate the principle of data minimization. In addition, doing so would create major security and privacy risks”. Inspired by the contributions made by the European Data Protection Board (2020) and European eHealth Network (2020), on 16th April 2020 the European Commission published COVID-europe 2020) the guidance on apps supporting the fight against COVID-19 pandemic in relation to data protection to ensure a coherent approach across the EU and provided guidance to Member States and app developers regarding the features and requirements that contact tracing apps should meet to ensure compliance with the EU privacy and personal data protection legislation, in particular the General Data Protection Regulation (GDPR) 2016) and the ePrivacy Directive (2002). In a very similar effort, on 4th May 2020 the UK Information Commissioner’s Office published (2020) data protection expectations on contact tracing app development outlining nine data protection principles (transparency, data minimization, user control, data security, etc.) which are linked to the core principles and provisions of data protection law and are designed to support design and development decisions of app developers.

The call for compliance with data protection acts and ethics is not only limited to the EU as it is globally demanded. On 28th May 2020, the World Health Organization (WHO) published an interim guideline covering the main ethical principles and requirements to achieve equitable and appropriate use of digital contact tracing technology. Again, most of these principles (transparency, data minimization, data retention, data security, etc.) are well-aligned with other globally endorsed data protection principles (WHO 2020). In this paper, we aim at inspecting the COVID-19 contact tracing apps from an information security and privacy perspective, to investigate if the developers and producers of these apps are mindful of the aforementioned legal requirements and if they have made their apps operational under privacy and security considerations aiming at respecting individuals’ privacy. For this reason, we examine available COVID-19 contact tracing apps for cues about their privacy and security quality through several lenses as detailed in the next sections.

## Study design

**Fig. 1:**
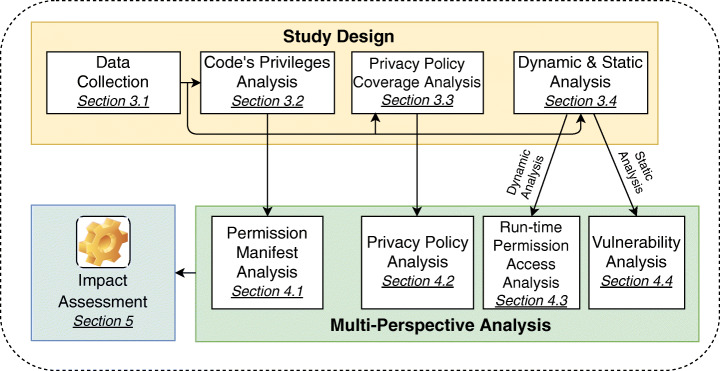
A high-level overview of multi-perspective privacy and security analysis of COVID-19 contact tracing apps

Our multi-perspective analysis comprises multiple static and dynamic analysis techniques enabling a comprehensive understanding of privacy and security performance of existing COVID-19 contact tracing apps. A high-level overview of our study is shown in Fig. 1. Our study consists of three main building blocks, namely *Study Design* (Section 3), *Multi-Perspective Analysis* (Section 4), and *Impact Assessment* (Section 5). The *Study Design* building block details the methods and design steps that were used as multiple inputs for the *Multi-Perspective Analysis*. As it is depicted in Fig. 1, the first lot of *Study Design* is data collection (Section 3.1). The second lot (code’s privileges analysis, Section 3.2) enabled an inspection of permission manifest (see Section 4.1). The third lot (privacy policy coverage analysis, Section 3.3) was subjected to inspection about policy coverage (see Section 4.2). Furthermore, the fourth lot (dynamic and static analysis, Section 3.4) was used to perform dynamic and static performance analyses (see Sections 4.3 and 4.4, respectively). It should be noted that the dynamic analysis phase yielded a secondary data set consisting of apps’ run-time permission access logs, which was populated through a one week data collection campaign in Germany and Sweden (elaborated in Section 4.3). We archived all the data sets in a Git Repository^2^. The *Multi-Perspective Analysis* building block serves as an input for the *Impact Assessment* aiming at synthesising the results obtained from our multi-perspective analysis. Since strong security and strong privacy are preconditions for designing IT products (including smartphone apps) (Cavoukian 2010), the methodology used in this paper relies on both privacy and security performance analysis of apps that avoids the pretense of false dichotomies such as privacy versus security. Hence, our work remains within the intersection of security and privacy aiming at investigating privacy and security quality indicators inspired by legal requirements such as data minimization, transparency, purpose limitation, and confidentiality. In addition, since our methodology comprises both static and dynamic analyses, it retains the main benefits of both techniques (Chaulagain et al. 2020; Choudhary and Kishore 2018). In what follows, we explain the steps that were taken to collect our data. Afterwards, we elaborate on the main pillars of the study design – as shown in Fig. 1.

### Data collection

Our study is focused on Android contact tracing apps, however, Google Play Store does not provide any specific category for these apps. We identify them by searching for the strings like “*covid contact tracing*”, “*covid*”, and “*contact tracing*” on Google Play Store’s search engine. However, such a search technique has two main limitations: (1) depending on the location where the queries are done (Germany and Sweden where the researchers of this paper were located), Google may eliminate some of the search results; and (2) such search strings sometimes return irrelevant results, e.g. COVID-19 symptom checker apps (that are not in the scope of this paper). Therefore, both these processes can produce false negatives and false positives, respectively. To overcome these limitations, for (1) we also repeated the same procedure on unofficial app stores (APKMirror 2020; APKPure 2020) that are location-independent that contain the APK files of apps regardless of where the search queries are done. For (2), we manually eliminate any app that does not introduce contact tracing (tracking and monitoring the spread of the virus) as one of its core functionalities. Furthermore, during our data collection process, we noticed that some of the contact tracing apps are not yet available in any app market other than their own websites (e.g. by regularly checking the list of produced COVID-related apps all over the globe (Covid-19 apps 2020)). As such, we were able to find and collect the APK files for those apps as well. In total, we found 28 contact tracing apps as shown in Table. 1.

### Code’s privileges

Mobile operating systems follow certain mechanisms to control and limit the amount of personal information accessed by apps (Hamed and Ben Ayed 2016). As a particular example, in Android, apps can request to access the device’s resources through permissions. Depending on the resource types, consent from users is required (Hatamian et al. 2017).

Every Android app has an AndroidManifest.xml file that contains information about that particular app (e.g., its name, author, icon, and description) and permissions that grant access to data such as call logs, contact lists or location tracks on smartphones (Momen et al. 2019). Approval from the user for granting a dangerous permission is required during the first use of the app. In such a permission managing scheme, it is difficult to perceive consequence for granting access and assess the risk, if not impossible (Zhauniarovich and Gadyatskaya 2016). Moreover, information is hardly available about the usage of permissions that are allowed access to resources (Hatamian et al. 2017). Hence, an app should only request and access those permissions that are relevant to its functionality. As such, this pillar of our analysis (see Section 4.1) collects and analyzes the code’s privileges, i.e. access intentions from the Android apps’ manifest to investigate the mapping, diversity, and critical aspects of COVID-19 contact tracing apps’ permission requests with respect to users’ privacy.

### Privacy policy coverage

The privacy policy of an app is a statement, or a legal document that gives information about the ways an app provider collects, uses, discloses, and manages users’ data. By law, service providers (including app providers) are required to be transparent about their data collection, sharing, and processing practices and specify how they comply with legal principles (Hatamian 2020). Moreover, privacy policies are the main sources that enable users to understand how their data is being handled by app developers/providers (Reidenberg et al. 2015). Hence, this pillar of our analysis (see Section 4.2) provides insights into the extent to which the privacy policy texts of COVID-19 contact tracing apps cover fundamental privacy policy principles. Our study focuses on the fulfillment of fundamental legal principles proposed in Hatamian (2020), the extent to which the privacy policy texts of COVID-19 contact tracing apps are correlated with what developers request (in manifest) and what they do in reality (actual permission usage), and ultimately the discrepancies/similarities in apps’ privacy policies published by the EU and non-EU bodies in terms of covering fundamental privacy principles. Based on keyword- and semantic-based search techniques, for each privacy policy a group of three data protection experts with legal and technical background of data privacy and security went through the texts. The goal was to figure out if they can find any overlap between each and every section of privacy policy texts and the legal principles discussed in the following. As our research is conducted in EU countries, our analysis is solely based on the GDPR. In the following, we briefly discuss each of the privacy policy principles.

#### Data Collection

The legal foundation is set in Art. 5 (1) and Art. 6 GDPR. While the former article states the general principles of processing personal data, the latter indicates when processing is lawful, including when consent is given, when it is necessary for the performance of a contract or compliance with a legal obligation, to protect vital interests of user or another natural person, and when processing is necessary for a task carried out in the public interest or for legitimate interests pursued by the controller or by a third-party. However, this applies if and only if such interests do not override the interests or fundamental rights and freedoms of users. Monetizing purposes, i.e., advertising, are not classified as necessary and therefore need to be based on another legal ground. Similarly, the processing of data to develop new features and services is not specific enough to comply with this section (ENISA 2017).

#### Children Protection

Information related to children must be treated with the utmost caution, as children “may be less aware of the risks, consequences, and safeguards concerned and their rights in relation to the processing of personal data” (Rec. 38 GDPR). This implies that services targeted at children are obliged to provide information in clear and plain language that children can understand easily (Rec. 58 GDPR). Art. 8 GDPR defines that the processing of children’s data is only lawful where the child is at least 16 years old. The data processing of younger children is only legitimate if and to the extent, a parent or legal guardian has given consent. However, this article has an opening clause, allowing member states to set a lower age for those purposes, yet not below 13 years.

#### Third-Party Sharing

Third-party components (that might collect data as well) are often integrated into an app’s development phase. The legal basis lies in Art. 13 (1e) GDPR, stating that the recipients or categories of recipients of personal data must be revealed to users.

#### Third-Country Sharing

The GDPR dedicates its *Chapter 5* to provisions on transfers of personal data to third-countries or international organizations. The transfer of data to other countries is only lawful, where a similar level of protection as provided by the GDPR is guaranteed. In fact, the protection of data travels with the data itself. Thus, if app providers share personal data with servers located outside the EU, they shall mention it in their privacy policy text how they deal with third-country data sharing practices.

#### Data Protection

The GDPR in Art. 32 states that the data controller must implement appropriate technical and organizational measures to ensure appropriate security. This is of particular importance in smartphone ecosystems since they are typically linked to a huge amount of data transfer. The aspect of data protection is also closely correlated with privacy-by-design (Cavoukian 2010).

#### Data Retention

The retention of data is a delicate issue, as app providers may want to retain data as long as possible to enable future transactions and purposes. However, this is often not in the interest of users, particularly not for sensitive data as available in smartphones (e.g., personal information from dating apps or health data from contact tracing apps). To protect users, the principle of data minimization and storage limitation in combination with transparency take effect. Accordingly, Art. 13 (2), 14 (2) of the GDPR state that the data controller must inform users for what period their data is retained. This is strictly required as users have “the right to be forgotten”, which is set in Art. 17 of the GDPR.

#### User’s Control

The whole *Chapter 3* of the GDPR is dedicated to the rights of users. The most important rights are the right to information and access to personal data; the right to rectification; the right to erasure (see the previous principle); the right to restriction of processing; the right to data portability; and the right to object and automated individual decision-making. By Art. 13 (2), 14 (2) of the GDPR, app providers are required to provide these rights to users to ensure fair and transparent data processing (principle of lawfulness, fairness and transparency Art. 5 (1a)).

#### Privacy Policy Changes

To further ensure lawful, fair, and transparent processing of data, app providers should inform users in a transparent and understandable way about privacy policy changes. This obligation is derived from Art. 12 of the GDPR.

#### Privacy Breach Notification

Besides Art. 12 GDPR, which lays the basis of informing users, this principle is based on Art. 34 GDPR where it is stated that if a data breach occurs that results in a high risk to the rights and freedoms of users, the data controller must inform users without undue delay. In this notification, the data protection officer must be named and likely consequences of the data breach as well as the measures taken to mitigate the effects are described. The same is applicable for the notification of the supervisory authority, which must be done not later than 72 hours after the detection of a personal data breach.

#### App-Focused

This principle is subsumed under the principle of lawfulness, fairness, and transparency. Sometimes a privacy policy is not exclusively written for a specific app, but multiple services provided by the same app developer (data controller). For instance, Sunyaev et al. (2015) identified five reoccurring scopes of privacy policies, namely privacy policies for a single app, for multiple apps, for a back-end app, for a developer homepage or for all developer services. They also found that several privacy policies of apps did not have an app-related scope at all.

#### Purpose Specification

This principle is closely related to the data collection principle. While the focus of data collection is on what data is collected, this principle refers to the clear statement of data collection purposes. Besides the legal basis for data processing, app providers are required to specify data collection purposes according to Art. 13 (1c), 14 (1c) GDPR. This is not only important under the aspect of lawfulness, fairness, and transparency, but also the principle of purpose limitation to prevent exploitation of personal data for other use cases.

#### Contact Information

Contact information is linked to the principle of lawfulness, fairness, and transparency. According to Art. 13 (1a), 14 (1a) GDPR, users have the right to be informed about the actual identity of data collectors, i.e., app providers. This includes the name of the app provider, if it is a legal entity, its legal representatives as well as its postal address. The latter must be provided to give users the possibility to file a formal complaint.

### Dynamic and static analysis

Mobile app users trade their data for service usage in opaque ways. Accessibility to user data through permissions gives *carte-blanche*^3^ access for an app without any constraints. Though the user has the option to revoke granted permissions, the absence of monitoring tools and unexpected consequences such as service exclusion (being unable to use a certain service) or malfunctions (e.g. UI malfunctioning) may cause hindrances in limiting access to permissions (Almuhimedi et al. 2015; Franzen and Aspinall 2016; Van Kleek et al. 2017). As such, it is not only important to assess the real data access patterns of apps, e.g. what personal data an app is accessing while the user is not using it, but also how the app performs in terms of limiting/minimizing potential vulnerabilities within its code. Therefore, this pillar of our analysis analyzes the contact tracing apps from two dimensions:

1. We dynamically measure apps’ real permission access patterns (see Section 4.3) based on our previously implemented tools described in Hatamian et al. (2018) and Momen (2018). Our approach is based on AppOps which is a privacy manager tool and introduced in Android 4.3.^4^ In order to collect logs, a timer is sent to the PermissionUsageLogger service periodically. When it is received, the logger queries the AppOps service that is already running on the phone for a list of apps that have used any of the operations we are interested in tracking. We then check through that list and for any app that has used an operation more recently than we have already checked, we store the time at which that operation was used in our own internal log. These timestamps can then be counted to get a usage count. We argue that such an analysis can reveal apps’ behavior and its impact on individual privacy, because of the fact that data collection can be identified as the first step that could lead to potential privacy violation (Daniel 2006).
2. We statically analyze the contact tracing apps (see Section 4.4) and look for potential vulnerabilities in their program codes using Mobile Security Framework (MobSF) (Mobile security framework (mobsf) 2020). MobSF is a security analysis tool that is capable of performing both static and dynamic analysis, penetration testing and malware analysis of Android, iOS and Windows apps. Thus, this pillar of our analysis examines the existing security vulnerabilities of COVID-19 contact tracing apps.

## Multi-perspective privacy and security analysis

In this section, we expand each pillar of our analysis method. Section 4.1 details the permission manifest analysis. Section 4.2 elaborates on the privacy policy coverage through inspecting apps’ privacy policy documents with respect to legally binding privacy principles. While Section 4.3 details the examinations regarding the real data access patterns of apps in a dynamic way, Section 4.4 digs into the steps that we took to uncover the potential vulnerabilities found in apps’ codes.

### Permission manifest analysis

In Android, apps can request access to the device’s resources through permissions. Depending on the resource types, consent from users is required. Android defines three types of permissions:^5^*normal*, *dangerous*, and *signature*. *Normal* level permissions allow access to resources that are considered low-risk, and they are granted during the installation of any package requesting them. The *dangerous* level permissions grant access to resources that are considered to be high-risk. In this case, the user is explicitly asked to grant permissions. So-called *signature* level permissions are granted at install time, but only when the app that attempts to use a permission is signed by the same certificate as the app that defines the permission. Every app has an AndroidManifest.xml file that contains information about that particular app (e.g., its name, author, icon, and description) and permissions that grant access to data such as call logs, contact lists or location tracks on smartphones. We collect and analyze app developers’ data access intentions from the Android apps’ manifest to investigate permission request patterns, suspicious permission requests, and discrepancies/similarities in apps’ permission requests published by EU and non-EU bodies.

#### Detected permission requests

Table 2 lists the detected permission requests by the apps within our data set together with their descriptions. In total, 31 permission requests were detected. Among these 31 permissions, 16 permissions (51.6%) belong to normal, 11 (35.4%) to dangerous, and 4 (12.9%) to signature categories, respectively.

**Table 2:** List of detected permissions within the data set and their descriptions

Type	Permission	Description
Normal	INTERNET	Allows to open network sockets.
Normal	BLUETOOTH	Allows to connect to paired bluetooth devices.
Normal	BLUETOOTH_ADMIN	Allows to discover and pair bluetooth devices.
Normal	FOREGROUND_SERVICE	Allows the use of foreground services.
Normal	REQUEST_IGNORE_BATTERY_OPITIMIZATION	Controls an app’s execution at the potential expense of the user’s battery life.
Normal	ACCESS_NETWORK_STATE	Allows to access information about networks.
Normal	WAKE_LOCK	Allows to keep processor from sleeping or screen from dimming.
Normal	RECEIVE_BOOT_COMPLETED	Allows the app to have itself started as soon as the system has finished booting.
Normal	VIBRATE	Allows access to the vibrator.
Normal	ACCESS_WIFI_STATE	Allows access to information about Wi-Fi networks.
Normal	CHANGE_WIFI_STATE	Allows to change Wi-Fi connectivity state.
Normal	READ_SYNC_SETTINGS	Allows to read the sync settings.
Normal	WRITE_SYNC_SETTINGS	Allows to write the sync settings.
Normal	BIND_GET_INSTALL_REFERRER_SERVICE	Allows to recognize where the app was installed from.
Normal	CHANGE_NETWORK_STATE	Allows to change network connectivity state.
Normal	MODIFY_AUDIO_SETTINGS	Allows to modify global audio settings.
Dangerous	ACCESS_FINE_LOCATION	Allows to access precise location.
Dangerous	ACCESS_COARSE_LOCATION	Allows to access approximate location.
Dangerous	ACCESS_BACKGROUND_LOCATION	Allows to access location in the background.
Dangerous	CALL_PHONE	Allows to initiate a phone call without user’s confirmation.
Dangerous	CAMERA	Allows access to the camera.
Dangerous	ACTIVITY_RECOGNITION	Allows the recognition of physical activities, e.g. user’s step count.
Dangerous	READ_EXTERNAL_STORAGE	Allows to read from external storage.
Dangerous	WRITE_EXTERNAL_STORAGE	Allows to write to external storage.
Dangerous	READ_PHONE_STATE	Allows read access to phone state, e.g. phone number and current cellular network information.
Dangerous	RECORD_AUDIO	Allows to record audio.
Dangerous	READ_CONTACTS	Allows to read the user’s contacts data.
Signature	GET_TASKS	Allows to discover information about which apps are used on the device.
Signature	SYSTEM_ALERT_WINDOW	Allows to create windows shown on top of all other apps.
Signature	PACKAGE_USAGE_STATS	Allows to collect component usage statistics.
Signature	REQUEST_INSTALL_PACKAGES	Allows to request installing packages.

#### Permission requests per app

**Fig. 2:**
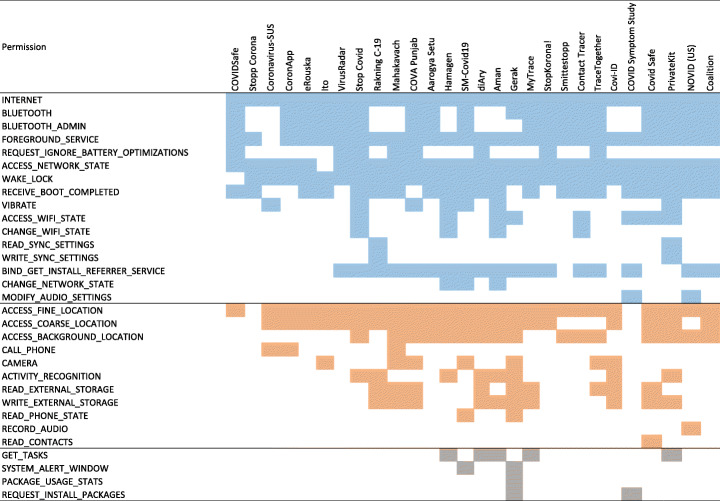
Details of permission requests per app: normal permissions (blue), dangerous permissions (orange), and signature permissions (gray)

Figure 2 shows the details of permission requests per app. Overall, there are 335 permission request incidents. Almost one-third (31%) of these incidents pertain to the category dangerous having a direct impact on users’ privacy, 66% normal permissions, and 3% signature permissions. Among the apps within our data set, Gerak and Mahakavach declare 8 (out of 18) and 7 (out of 14) of their permission requests from dangerous permission category, respectively. Followed by this, each of diAry, Covid Safe, and COVID Punjab apps requests 6 permissions from dangerous category. By contrast, we also found apps in our data set that either do not ask for any dangerous permission (1 app, Stopp Corona) or only ask for one dangerous permission (COVIDSafe). Figure 2 clearly indicates that, in general, COVID-19 contact tracing apps require a mixed variety of permissions to provide their desired services. In terms of median value, COVID-19 contact tracing apps request 12 permissions, 4 of them being labeled as dangerous.

#### Permission type analysis

**Fig. 3:**
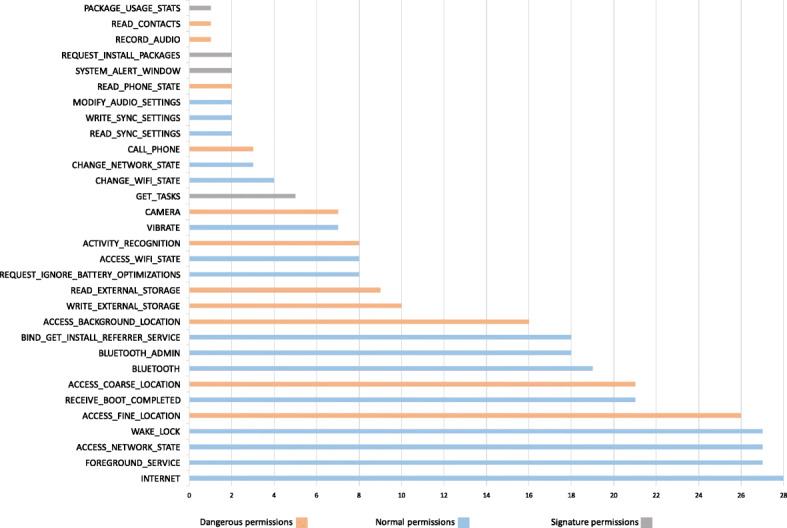
Permissions found from the manifest analysis: the most sought after dangerous permission is ACCESS_FINE_LOCATION (found in 26 apps out of 28), and READ_CONTACTS and RECORD_AUDIO are requested by only one app

Figure 3 shows the commonly found permissions within our data set based on their permission type. The analysis shows that dangerous permission requests accounted for 30% of the top 10 requested permissions, namely ACCESS_FINE_LOCATION (26 apps), ACCESS_COARSE_LOCATION (21 apps), and ACCESS_BACKGROUND_LOCATION (16 apps). Furthermore, when it comes to dangerous permission requests, the contact tracing apps indicate a hungriness level of requesting the following permissions: ACCESS_FINE_LOCATION (26 apps), ACCESS_COARSE_LOCATION (21 apps), ACCESS_BACKGROUND_LOCATION (16 apps), WRITE_EXTERNAL_STORAGE (10 apps), READ_EXTERNAL_STORAGE (9 apps), ACTIVITY_RECOGNITION (8 apps), CAMERA (7 apps), CALL_PHONE (3 apps), READ_PHONE_STATE (2 apps), RECORD_AUDIO (1 app), and READ_CONTACTS (1 app).

Although our main focus is on dangerous permission requests, non-dangerous permission requests can also pose serious risks on users’ privacy. It is important to highlight that non-dangerous permissions can be easily misused to profile users. As a particular example, GET_TASKS permissions which is requested by 5 apps can reveal sensitive information about which apps are being used by the user. As a result, the provider of the contact tracing apps is able to see ActivityManager.RecentTaskInfo^6^ that can retrieve information about tasks that the user has most recently started or visited. Similarly, SYSTEM_ALERT_WINDOW is another example of non-dangerous permissions that can reveal highly sensitive information through creating overlays that can trick users by covering certain areas of the screen while making the overlaid area responsive (Alepis and Patsakis 2017, 2019).

It is also important to highlight that the combination of such permission requests may reveal interesting information about users (Fritsch and Momen 2017; Momen and Fritsch 2020). For example, ACCESS_WIFI_STATE (requested by 8 apps), CHANGE_NETWORK_STATE (requested by 3 apps), and CHANGE_WIFI_STATE (requested by 4 apps) – these permissions are automatically granted to the apps as they are labeled as normal. The combination of accessing them allows an app to connect and disconnect from a given WiFi network. Such a combination of permission requests allows an app to retrieve information of the connected WiFi networks which can reveal highly sensitive information such as how long certain users stayed in a similar proximity, how often, when exactly, etc. Achara et al. (2014) and Alepis and Patsakis (2019).

#### The GDPR impact

**Fig. 4:**
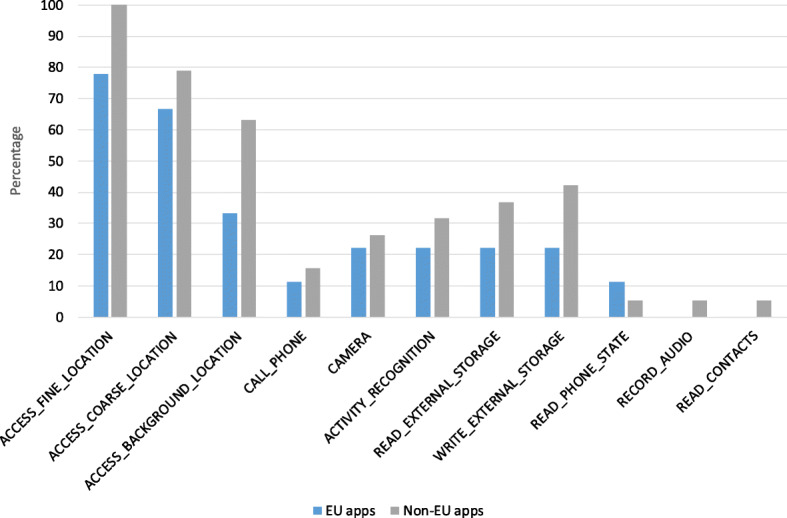
Comparison of dangerous permissions found in the manifest analysis: EU vs. non-EU contact tracing apps (the lower, the better)

As it can be observed from our data set discussed in Section 3.1, the studied contact tracing apps have been developed and published across different countries. Therefore, this aspect of our analysis deals with the question of how contact tracing apps may behave differently or similarly in terms of dangerous permission requests when it comes their geographic area in which they got produced and published. Since our research is conducted in Europe and the legal requirements enforced by the EU General Data Protection Regulation (GDPR) (2016) and relevant European authorities serve as a benchmark for our multi-perspective privacy and security analysis, we generally categorize all the apps within our data set into two main categories, namely *EU* (9 apps) that mainly covers those Member States where the GDPR is enforced and *non-EU* apps (19 apps). Such a comparison enables us to not only compare the behavior of certain apps within a category, but also the discrepancies or similarities between each individual category in terms of requesting dangerous permissions. Figure 4 shows the comparative analysis in terms of dangerous permission requests per category (percentage is calculated by dividing the number of apps in a certain group, e.g. non-EU apps, which request a certain dangerous permission by the total number of apps in that group). Overall, the results clearly indicate that the developers/providers of apps published within the EU category request less sensitive permissions than others. One potential interpretation for such a behavior might be the strong enforcement of the EU GDPR along with other strict European guidelines concerning designing and developing contact tracing apps (COVID-europe 2020, 2020, 2020). Except for phone-related information (e.g. IMEI and phone number), the EU apps request less sensitive permissions than non-EU apps in all respects. For instance, when it comes to location-related permission requests, non-EU apps request slightly more than double in comparison to the EU ones. The reason for such behavior may be threefold. Firstly, the GDPR has a clear and strict vision on data minimization and purpose specification as it states in its Art. 5 (1b) that personal data shall be “collected for specified, explicit and legitimate purposes and not further processed in a manner that is incompatible with those purposes; further processing for archiving purposes in the public interest, scientific or historical research purposes or statistical purposes shall, in accordance with Art. 89 (1), not be considered to be incompatible with the initial purposes”. Further, it states (in Art. 6 (4)) that “data processing for incompatible purposes should be avoided unless it is on the basis of a specific set of criteria in the GDPR”. Secondly, in the EU, collecting location data forever and obtaining single user consent (for multiple purposes) was unacceptable under the previous Data Protection Directive (Directive 95/46/EC) (1995). Nevertheless, thanks to the GDPR, it is now even more stricter as the GDPR provides more detailed information on data usage and retention and consent becomes even more specific. Thirdly, ePrivacy Directive (2002) more in details elaborate on the issue of location data collection and clearly states that such a data collection may result in high privacy risks, particularly when individuals’ movement patterns are tracked. Further, it states that “such data may only be processed when they are made anonymous, or with the consent of the users or subscribers to the extent and for the duration necessary for the provision of a value added service. The service provider must inform the users or subscribers, prior to obtaining their consent, of the type of location data other than traffic data which will be processed, of the purposes and duration of the processing and whether the data will be transmitted to a third party for the purpose of providing the value added service. Users or subscribers shall be given the possibility to withdraw their consent for the processing of location data other than traffic data at any time”. Nevertheless, we should highlight that although the EU apps have better performance (compared to non-EU apps) in asking location-related permissions, their behavior is still not compliant with the requirements and recommendations published by the European Commission and other EU data protection authorities as many of them are asking to access such data.

### Privacy policy analysis

In our analysis, we pay attention to privacy policy analysis of contact tracing apps and fulfillment of 12 fundamental legal principles proposed in Hatamian (2020), the extent to which the privacy policy texts of COVID-19 contact tracing apps match what developers request (in manifest) and what they do in reality (actual permission usage), and ultimately the discrepancies/similarities in apps’ privacy policies published by EU and non-EU bodies in terms of covering fundamental privacy principles. However, one should bear in mind that since these principles are extracted from the GDPR, they may not be necessarily applicable to all non-EU apps, especially those that do not offer a strong data protection regulation as offered by the GDPR.

#### Privacy policy completeness analysis

**Fig. 5:**
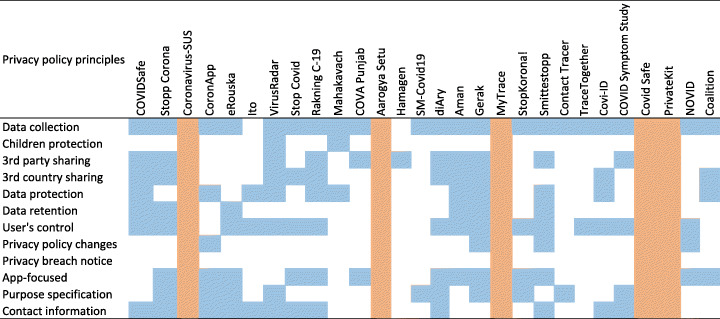
Details of privacy policy principles fulfillment per app. We differentiate principle fulfillment (blue), from non-fulfillment (white), and absence of privacy policy text or very generic/outdated text (orange)

Figure 5 shows the results of privacy policy analysis of the apps within our data set. The result indicates to which extent an app has been successful to fulfill the privacy policy principles. The results show that the Gerak app fulfills the maximum number of principles (9 principles), followed by Stopp Corona (8 principles) and VirusRadar (8 principles). Surprisingly, our findings also revealed that some of these apps (five apps) do not fulfill any privacy policy principle either because they do not have any privacy policy text accessible on the Internet that is the case for two of them (e.g. Coronavirus-SUS and Aarogya Setu) or because they have very generic text that does not discuss the data collection and sharing practices of apps, rather irrelevant information that is the case for three of them (MyTrace, Covid Safe, and PrivateKit).

**Fig. 6:**
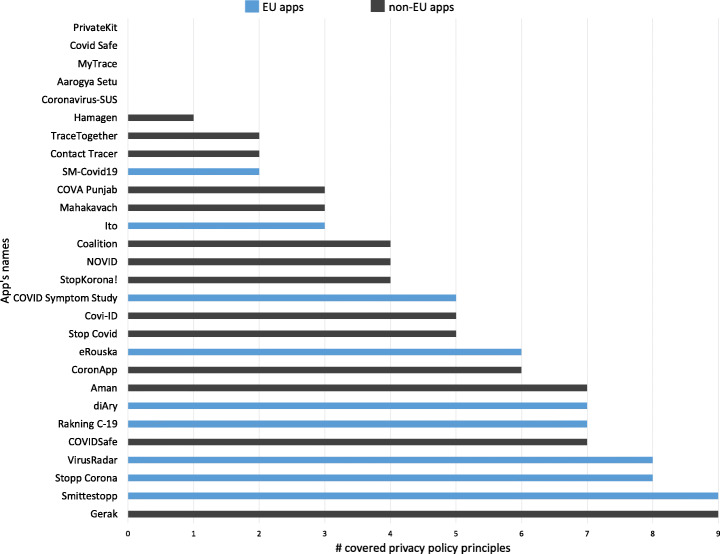
Total number of privacy policy principles covered by each app

Figure 6 provides a detailed overview regarding the total number of privacy policy principles covered by each contact tracing app. As it can be seen, the maximum number of principles covered is 9 (out of 12). Moreover, only 10 (35.7%) apps covered more than half of the principles, and the rest either did not cover any principles (17.8%, 5 apps) or covered less than half of the principles (46.5%, 13 apps).

#### Coverage of privacy policy principles

**Fig. 7:**
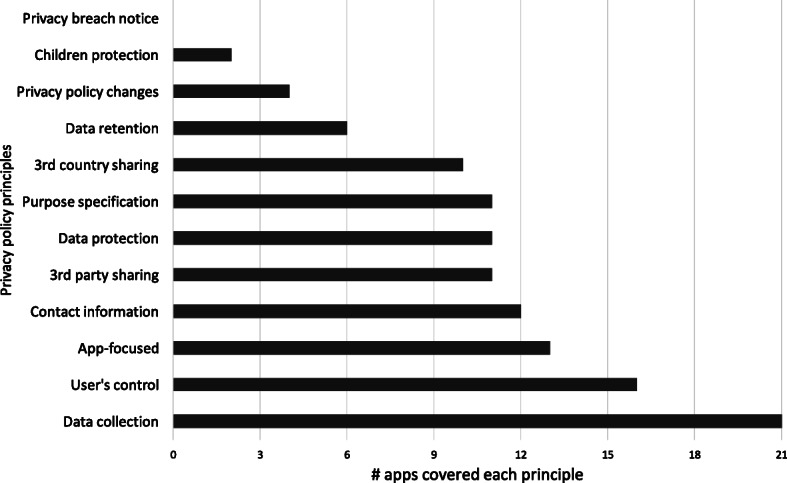
Privacy policy principles coverage

Figure 7 shows the coverage of privacy policy principles by the apps within our data set. Our inspection shows that *data collection* is the most covered principle (17 apps). Surprisingly, we found out that no apps fulfilled the *privacy breach notice* principle. This is highly critical as service providers in EU are enforced by law to adopt appropriate remedies in case of a data breach. This also shows that none of these apps are well-prepared in case users’ personal data fall into the wrong hands due to a privacy breach. The same also holds for *children protection* and *privacy policy changes* where only a few apps (1 app for *children protection* and 3 apps for *privacy policy changes*) clarified how their data collection, sharing, and processing practices fulfill these essential principles.

#### Dangerous permission transparency in privacy policy document

**Fig. 8:**
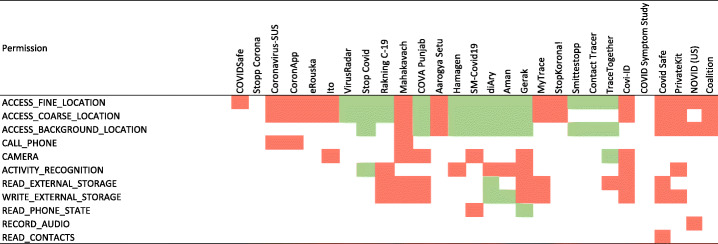
Details of dangerous permission usage transparency in privacy policy text of contact tracing apps. We differentiate permission usage transparency (green), from permission usage non-transparency (red)

Transparency is a basic data protection principle endorsed by privacy-by-design (Cavoukian 2010) and the GDPR. Importantly, it is one of the fundamental principles strictly endorsed by the WHO (2020) to be followed by the developers of contact tracing apps. Therefore, it is of particular importance to examine the extent to which contact tracing apps fulfill such a requirement. We developed a set of relevant keywords (e.g. *location*, *proximity*, *precise*, *approximate*, *track*, *movement*, *gps*, and so on) corresponding to each dangerous permission (e.g. ACCESS_FINE_LOCATION) defined by Android^7^ to conduct a manual observation for this task. As shown in Fig. 8, we found that only 14.2% (4 apps) of contact tracing apps fully justify the need for requesting dangerous permission requests. Further, 28.5% (8 apps) of them only partially clarify why they need to access certain dangerous permissions. This indicates that more than half of the contact tracing apps (57.2%, 16 apps) failed to specify the need for requesting dangerous permissions.

#### The GDPR impact

**Fig. 9:**
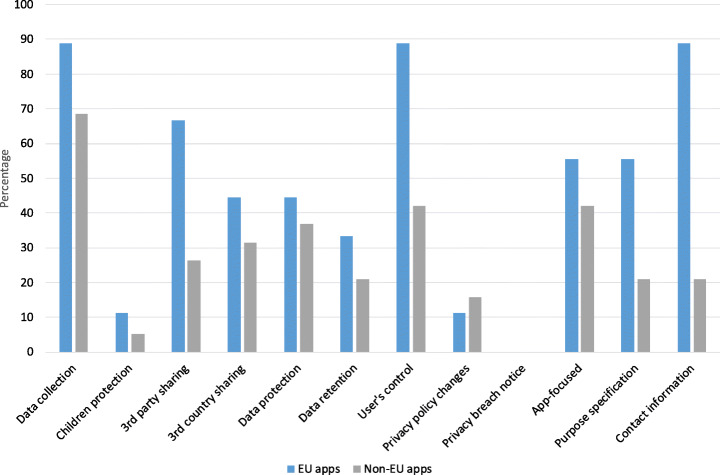
Privacy policy performance: EU vs. Non-EU contact tracing apps (the higher, the better)

We also conducted a comparative analysis similar to the analysis that we carried out regarding the permission requests of apps across the EU and rest of the world to compare the privacy policy principles fulfillment of contact tracing apps developed and published by EU and non-EU bodies. Figure 9 shows the comparative analysis in terms of privacy policy performance per category. Overall, similar to what we observed concerning the permission manifest analysis, the EU apps are the most legal compliant ones. Except for *privacy policy changes*, the EU apps perform better in all respects. For instance, when it comes to *user’s controls*, only 42.1% of non-EU apps provide some specifications on how they allow users to exercise their rights (the percentage is calculated by dividing the number of apps in a certain group, e.g. non-EU apps, which fulfill a certain legal principle by the total number of apps in that group). While this percentage is 88.8% for European apps. As for *contact information*, only one-fifth of non-EU apps provided precise contact information in order to enable users (data subjects) to contact them. However, this amount is almost 90% for EU apps. The results clearly show that EU apps comply with the GDPR better than non-EU apps.

### Run-time permission access pattern analysis

Run-time analysis is another pillar of our multi-perspective analysis that provides us with information regarding the permission access patterns of contact tracing apps at run-rime. This is of particular importance as once a permission is granted to an app through the Android permission manager system that was introduced in API 23 in Android 6.0 (Marshmallow)^8^ to offer granular control to users, its risks are not fully mitigated (Fritsch and Momen 2017; Hatamian et al. 2017; Momen et al. 2017) as granted privileges remain available to resource-hungry apps and advertising libraries that could lead to privacy implications (Momen et al. 2019, 2020). This is why this section focuses on the question about how the apps exercise their granted privileges to access permissions at run-time.

#### Test-bed for monitoring permission usage

**Fig. 10:**
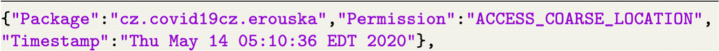
An example of collected logs by the monitoring tool

Permission usage monitoring was conducted using our previously proposed tools (Hatamian et al. 2018; Momen 2018) through logging, collecting, and analyzing permission access patterns of contact tracing apps (e.g. access to sensitive resources like GPS). To analyze the behavior of contact tracing apps listed in Table 1, we installed our monitoring tool together with these 28 apps on laboratory devices. Next, while our monitoring tool was running in the background the whole time (i.e., it was monitoring the permission access frequency of apps), we started to open each and every contact tracing app once to trigger and activate their desired functionality. To make sure that we did not miss any certain functionality, we deliberately granted permissions whenever asked by the apps (either through the GUI or the permission manager system of Android). Afterwards, we let the apps to execute in the background (without any further interactions, but were connected to WiFi network and a power source). This was by intention as our goal was to figure out if apps access any sensitive resources while there is no legitimate reason for that. After a one week ongoing experiment, the data generated by the monitoring tool was collected and analyzed. Since the researchers of this study were located in two EU countries (Sweden and Germany), the log collection campaigns were carried out simultaneously in these two countries to investigate if contact tracing apps behave differently when being used in different geographic locations. Our objective was to find out what is being accessed by apps, at what time, and at which frequency. For instance, Fig. 10 shows an example of collected logs, where eRouska accesses a location-related permission at a certain time.

#### Results of permission access pattern analysis

**Fig. 11:**
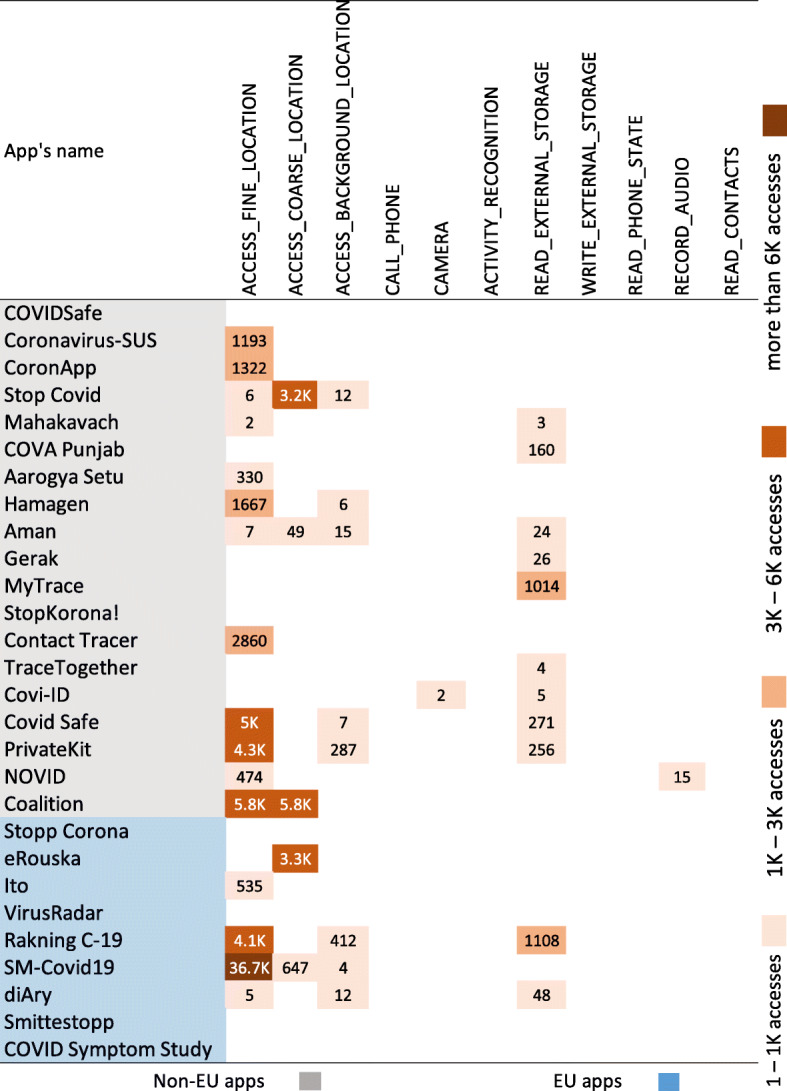
Run-time permission access analysis results

Our motivation behind running parallel data collection campaigns in Germany and in Sweden about apps’ run-time behavior was to identify geographical influence and to achieve clarity about app behavior. However, we observed similar, if not identical, permission access patterns during run-time. Hence, Fig. 11 presents a cumulative result from both data collection campaigns during a one week period. Majority of the apps (17 out of 28) are found to be accessing three variations of LOCATION permission. Hence, they create risks to location privacy that might lead to citizen tracking (Fritsch 2008b). Among them, 12 apps are from non-EU countries and 5 apps are from EU countries. Non-EU apps seem to be exercising location privileges more often. However, an EU app – SM-Covid19 (Italy) showed the highest number of permission access within one week of data collection period (36.7K), which cancels out the good behavior (fewer permission access counts) of other apps. Variants of STORAGE permission (cumulatively presented as READ_EXTERNAL_STORAGE) were accessed by 9 non-EU apps and by 2 EU apps. Other than these, 2 non-EU apps accessed CAMERA (Covi-ID) and RECORD_AUDIO (Novid).

#### App behavior: Timeline inspection

**Fig. 12:**
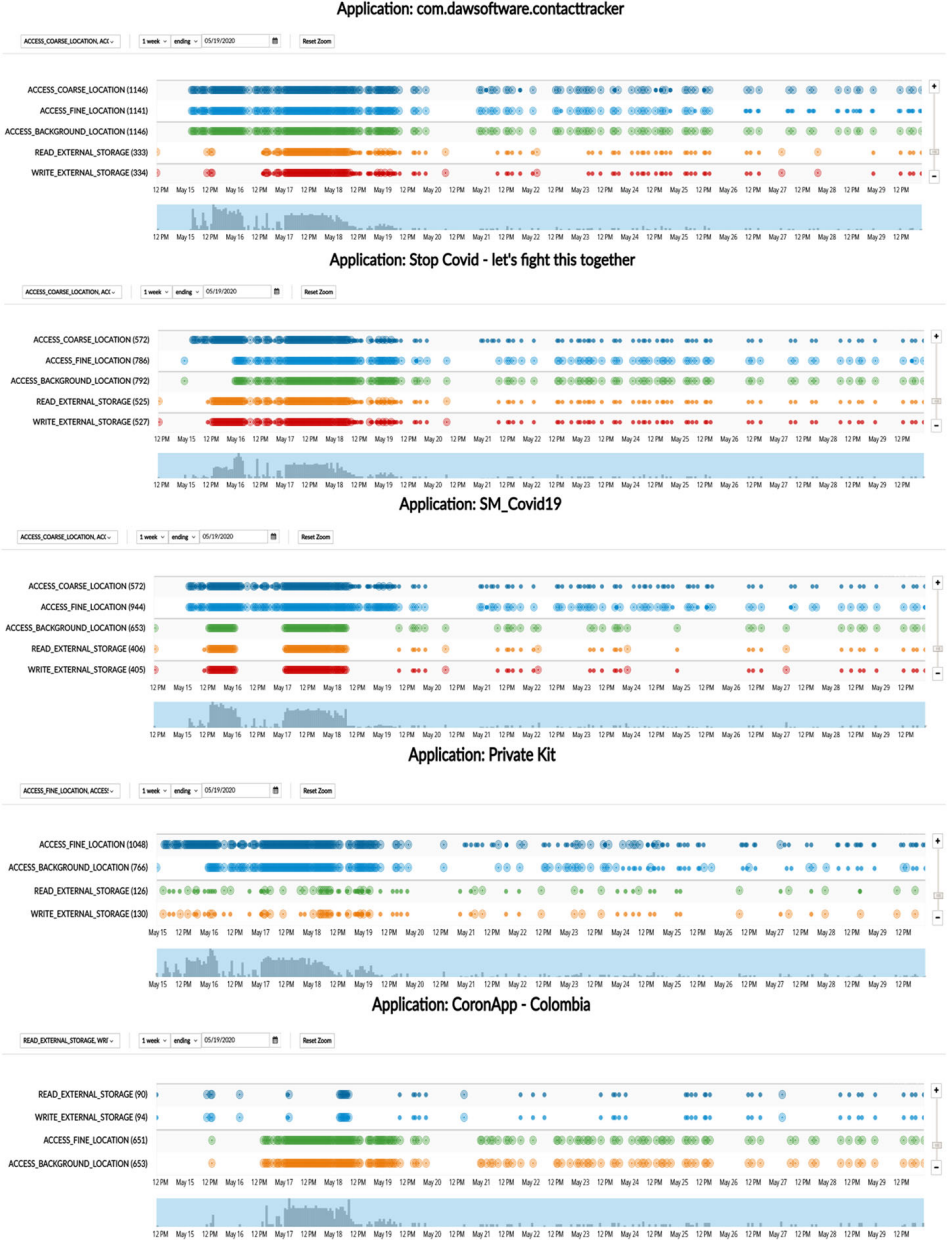
Timeline inspection of apps’ permission access pattern (dangerous permissions only): top five *privacy-invasive* permission access patterns

**Fig. 13:**
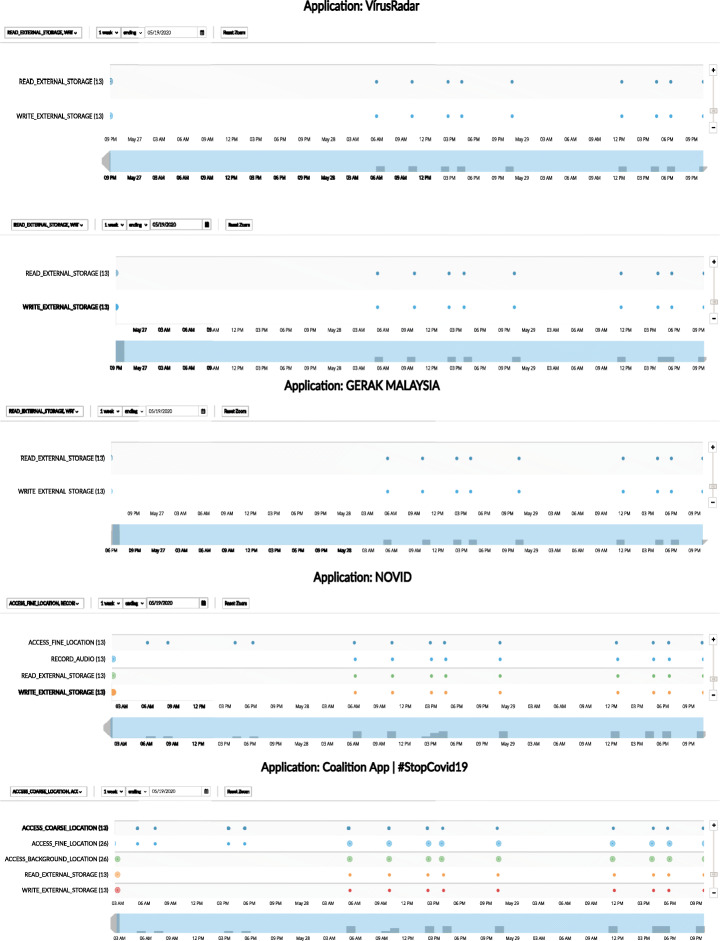
Timeline inspection of apps’ permission access pattern (dangerous permissions only): top five *privacy-preserving* permission access patterns

In Figs. 12 and 13, we present a timeline of the most and the least frequent permission access patterns observed in the data set from Sweden (top five and bottom five, respectively). Figure 12 presents the timeline of permission access patterns of the top five permission-hungry apps: (i) Contact tracer, (ii) Stop Covid, (iii) SM-Covid19, (iv) Private Kit, and (v) CoronApp. All of them are found to be accessing the location-related permissions frequently such as ACCESS_FINE_LOCATION. Figure 13 shows a visual timeline of the privilege access patterns of the top five privacy-preserving apps from our data set: (i) VirusRadar, (ii) Stopp Corona, (iii) Gerak Malaysia, (iv) Novid and (v) Coalition. Their permission access patterns demonstrate significantly fewer usage than the rest. However, Coalition showed permission access event for LOCATION permission, whereas, Novid showed permission access event for RECORD_AUDIO permission in the timeline (although this might be justifiable to a certain extent due to the app being relied on ultrasonic technology, we wonder if these accesses are legitimate due to the app not being used actively). It should be noted that our analysis suffers from the limitation of false positive in the indication of good behavior. Though permission access frequency is rather low for these five apps in Fig. 13, they could behave differently during real-life usage (e.g. with user interaction). On the other hand, the app could have over-privilege-issue, should it not require the corresponding permission. For example, good behavior is documented for both VirusRadar and Gerak in Fig. 13, and at the same time, Fig. 2 shows that they have the LOCATION permission listed in its manifest file. Similarly, Novid and Coalition have the potential to behave in a privacy invasive manner (in terms of permission access pattern) during real life usage. Among the top five privacy-preserving apps (according to their permission access pattern, as depicted in Fig. 13), only Stopp Corona does not have any dangerous permission listed in its manifest file.

What needs to be highlighted here is the fact that many apps access location-related permissions. We argue that this is extremely problematic from a privacy perspective due to four main reasons: (1) according to regulatory guideline documents (COVID-europe 2020; EDPB-letter 2020) contact tracing apps should not request and access location data (as this is not relevant to their proper functionality), (2) even though in some cases apps state (in their privacy policies) that they request location data, we believe this is still problematic and not aligned with best privacy and security practices, (3) as detailed in Fig. 8, many apps failed to state if they use a certain dangerous permission (including location-related ones) and many of them started to access it in a non-transparent manner (Coronavirus-SUS, Ito, Covid Safe, PrivateKit, etc.), and (4) we are aware that in Android, apps must declare location permission in order to be able to access Bluetooth^9^. However, this does not mean that apps that use Bluetooth can actively use location permission. Granting location permission is meant to activate the general system setting *Location Services*. We observed that some of these apps (e.g. Ito, Covid Safe, Coalition) claim to rely on BLE technology, but our analysis shows that they have been accessing location-related permissions.

### Vulnerability analysis

One of the essential and critical aspects highlighted by the eHealth Network (COVID-europe 2020) is the security of COVID-19 contact tracing apps. With the governments’ and developers’ rushing to roll out contact tracing apps, concerns are emerging in relation to the fact that there could be a broad range of security vulnerabilities lurking within these apps, which could put an individual’s sensitive information at risk of being exploited. Further, these apps access dangerous permissions (as highlighted in the previous sections), which suggests that they indicate potential security risks. Therefore, it is highly important that these COVID-19 apps are secured against malicious attacks as they access, store and transmit sensitive data.

In order to lessen any potential harm that would emerge from exploiting a vulnerability, an analysis is required in detecting potential security weaknesses that could be exploited and leveraged by attackers within these apps. As such, we applied static analysis to the complete app set shown in Table 1 using MobSF (Mobile security framework (mobsf) 2020). While there are other tools capable of performing static code analysis, MobSF was selected based on its popularity, scalability, easy adoption and its effectiveness in identifying vulnerabilities within Android apps much quicker (Ibrar et al. 2017; Zhang et al. 2018). Vulnerability assessment through static analysis relies on data contained in the app’s APK file, which includes the manifest and the compiled code (Knorr et al. 2015). In this case, however, we do not dwell on the permission analysis as it has been extensively discussed in the prior sections.

We analyzed the results generated by the MobSF with the intention of identifying critical vulnerabilities within the apps and comparing the security performance of apps published by EU and non-EU bodies.

#### Detected vulnerabilities

Tables 3 and 4 provide an overview of the security issues identified from the analysis of the APK files using MobSF. The framework uses Common Vulnerability Scoring System Version 2.0 (CVSS V2), Common Weakness Enumeration (CWE) and Open Web Application Security Project (OWASP) Top 10 Mobile Risks to list and score some of the common vulnerabilities within the app set as seen in Table 3, where the vulnerabilities identified (listed as issues) have been selected and matched against these standards. While each app has considerable amount of vulnerabilities as per the report generated by MobSF, we picked vulnerabilities that had a score ranging from medium to high as highlighted under the CVSS V2. CVSS V2 provides an open framework for communicating the characteristics and impacts of IT vulnerabilities (Mell et al. 2007) while CWE^10^ provides a point of reference for common vulnerabilities within software and hardware, and baseline for vulnerability identification. On the other hand, OWASP Top 10 provides a list of top ten mobile risks which creates awareness about application and software security (Qian et al. 2018). In addition to these, the framework provides the app’s average CVSS score and App Security Score.

**Table 3:** Summary of selected vulnerabilities within the COVID-19 app set

App	Issue	Standard		
		CVSS V2	CWE	OWASP
COVIDSafe	Files may contain hardcoded sensitive information like username, password, keys etc.	7.4 (H)	CWE-312 - Cleartext Storage of Sensitive information	M9 - Reverse Engineering
Stopp Corona	Uses SQLite Database and execute raw SQL query. Untrusted user input in raw SQL queries can cause SQL injection. Also, sensitive information should be encrypted and written to the database.	5.9 (M)	CWE-89 - Improper Neutralization of Special Element used in an SQL Command (’SQL Injection’)	M7 - Client Code Quality
Coronavirus-SUS	Uses SQLite Database and execute raw SQL query. Untrusted user input in raw SQL queries can cause SQL injection. Also, sensitive information should be encrypted and written to the database.	5.9 (M)	CWE-89 - Improper Neutralization of Special Element used in an SQL Command (’SQL Injection’)	M7 - Client Code Quality
CoronApp	Uses an insecure Random Number Generator.	7.5 (H)	CWE-330 - Use of Insufficiently Random Values	M5 - Insufficient Cryptography
eRouska	Uses an insecure Random Number Generator.	7.5 (H)	CWE-330 - Use of Insufficiently Random Values	M5 - Insufficient Cryptography
Ito	Uses SQLite Database and execute raw SQL query. Untrusted user input in raw SQL queries can cause SQL injection. Also, sensitive information should be encrypted and written to the database.	5.9 (M)	CWE-89 - Improper Neutralization of Special Element used in an SQL Command (’SQL Injection’)	M7 - Client Code Quality
VirusRadar	Uses an insecure Random Number Generator.	7.5 (H)	CWE-330 - Use of Insufficiently Random Values	M5 - Insufficient Cryptography
Stop Covid	Files may contain hardcoded sensitive information like username, password, keys etc.	7.4 (H)	CWE-312 - Cleartext Storage of Sensitive information	M9 - Reverse Engineering
Ranking C-19	Files may contain hardcoded sensitive information like username, password, keys etc.	7.4 (H)	CWE-312 - Cleartext Storage of Sensitive information	M9 - Reverse Engineering
Mahakavach	MD5 is a weak hash known to have hash collisions.	7.4 (H)	CWE-327 - Use of a Broken or Risky Cryptographic Algorithm	M5 - Insufficient Cryptography
COVA Punjab	Uses SQLite Database and execute raw SQL query. Untrusted user input in raw SQL queries can cause SQL injection. Also, sensitive information should be encrypted and written to the database.	5.9 (M)	CWE-89 - Improper Neutralization of Special Element used in an SQL Command (’SQL Injection’)	M7 - Client Code Quality
Aarogya Setu	Uses ECB mode in cryptographic encryption algorithm. ECB mode us known to be weak as it results in the same ciphertext for identical blocks of plaintext.	5.9 (M)	CWE-327 - Use of a Broken or Risky Cryptographic Algorithm	M5 - Insufficient Cryptography
Hamagen	Files may contain hardcoded sensitive information like username, password, keys etc.	7.4 (H)	CWE-312 - Cleartext Storage of Sensitive information	M9 - Reverse Engineering
SM-Covid19	Uses SQLite Database and execute raw SQL query. Untrusted user input in raw SQL queries can cause SQL injection. Also, sensitive information should be encrypted and written to the database.	5.9 (M)	CWE-89 - Improper Neutralization of Special Element used in an SQL Command (’SQL Injection’)	M7 - Client Code Quality
diAry	Files may contain hardcoded sensitive information like username, password, keys etc.	7.4 (H)	CWE-312 - Cleartext Storage of Sensitive information	M9 - Reverse Engineering
Aman	Files may contain hardcoded sensitive information like username, password, keys etc.	7.4 (H)	CWE-312 - Cleartext Storage of Sensitive information	M9 - Reverse Engineering
Gerak	Files may contain hardcoded sensitive information like username, password, keys etc.	7.4 (H)	CWE-312 - Cleartext Storage of Sensitive information	M9 - Reverse Engineering
MyTrace	MD5 is a weak hash known to have hash collisions.	7.4 (H)	CWE-327 - Use of a Broken or Risky Cryptographic Algorithm	M5 - Insufficient Cryptography
StopKorona!	Uses an insecure Random Number Generator.	7.5 (H)	CWE-330 - Use of Insufficiently Random Values	M5 - Insufficient Cryptography
Smittestopp	Uses SQLite Database and execute raw SQL query. Untrusted user input in raw SQL queries can cause SQL injection. Also, sensitive information should be encrypted and written to the database.	5.9 (M)	CWE-89 - Improper Neutralization of Special Element used in an SQL Command (’SQL Injection’)	M7 - Client Code Quality
Contact Tracer	Insecure implementation of SSL. Trusting all the certificates or accepting self signed certificates is a critical security hole. This app is vulnerable to MITM attacks.	7.4 (H)	CWE-295 - Improper Certificate Validation Cryptographic Algorithm	M3 - Insecure Communication
Trace Together	Remote Webview Debugging is Enabled.	5.4 (M)	CWE: CWE-919 - Weakness in Mobile Application	M1 - Improper Platform Usage
Covi-ID	MD5 is a weak hash known to have hash collisions.	7.4 (H)	CWE-327 - Use of a Broken or Risky Cryptographic Algorithm	M5 - Insufficient Cryptography
COVID Symptom Study	Files may contain hardcoded sensitive information like username, password, keys etc.	7.4 (H)	CWE-312 - Cleartext Storage of Sensitive information	M9 - Reverse Engineering
COVID Safe	Uses an insecure Random Number Generator.	7.5 (H)	CWE-330 - Use of Insufficiently Random Values	M5 - Insufficient Cryptography
PrivateKit	Files may contain hardcoded sensitive information like username, password, keys etc.	7.4 (H)	CWE-312 - Cleartext Storage of Sensitive information	M9 - Reverse Engineering
NOVID	Uses SQLite Database and execute raw SQL query. Untrusted user input in raw SQL queries can cause SQL injection. Also, sensitive information should be encrypted and written to the database.	5.9 (M)	CWE-89 - Improper Neutralization of Special Element used in an SQL Command (’SQL Injection’)	M7 - Client Code Quality
Coalition	Uses SQLite Database and execute raw SQL query. Untrusted user input in raw SQL queries can cause SQL injection. Also, sensitive information should be encrypted and written to the database.	5.9 (M)	CWE-89 - Improper Neutralization of Special Element used in an SQL Command (’SQL Injection’)	M7 - Client Code Quality

**Table 4:** An overview of the results from mobSF static analysis

Issue	Percentage of apps with the issues
The app logs information. Sensitive information should never be logged	100%
This app uses Java Hash Code. It is a weak hash function and should never be used in Secure Crypto Implementation	100%
Files may contain hardcoded sensitive information like username, password, keys etc.	89.3%
The App uses an insecure Random Number Generator	82.1%
App can write to App directory. Sensitive information should be encrypted	28.6%
IP Address Disclosure	57.1%
SHA-1 is a weak hash known to have hash collision	35.7%
The App uses SQLite Database and execute raw SQL query. Untrusted user input in raw SQL queries can cause SQL injection. Also, sensitive information should be encrypted and written to the database.	75%
App creates temp file. Sensitive information should never be written into a temp file.	28.6%
App can read/write to external storage. Any app can read data written to external storage	39.3%
MD5 is a weak hash known to have hash collisions	39.3%
Insecure Webview Implementation. Execution of user controlled code in WebView is a critical security hole	21.4%
The App uses ECB mode in cryptographic encryption algorithm. ECB mode us known to be weak as it results in the same ciphertext for identical blocks of plaintext	7.1%
Remote WebView debugging is enabled	7.1%
Insecure implementation of SSL. Trusting all the certificates or accepting self signed certificates is a critical security hole. This app is vulnerable to MITM attacks	3.6%

The outcome of the analysis revealed a number of vulnerabilities which could be considered as critical in this case. For example, 89.3% of the analyzed apps, apart from Stopp Corona (Austria), Novid (US) and Mahakavach (India), contain potentially hard-coded sensitive information like usernames, passwords, keys etc, which is considered to be of high severity as mapped under the CVSS V2 (with a 7.4 score); however, while the vulnerability does not match to a CWE indicated in Table 4, the use of hard-coded sensitive information (CWE-798) is of high risk as it could allow an attacker to circumvent authentication set by a software administrator.^11^ This information can be accessed through reverse engineering the contact tracing apps’ source code. As a result, armed with this information, an attacker is able to gain access to even more sensitive information, including the health status and location data.

Further analysis of the results indicate that 75% of the apps use SQLite Database and execute raw SQL query, apart from Contact Tracer, StopKorona!, COVIDSafe, CoronApp, eRouska, VirusRadar, and StopCovid. Untrusted user input in raw SQL queries can potentially lead to a local SQL Injection in the contact tracing app. In addition to this, the apps tend to use an unencrypted SQLite Database. This leaves the sensitive information lying open to attackers (Jain and Shanbhag 2012) with physical access to the mobile device or a malicious app with root access to the device. Besides this, lack of encryption could lead to privacy infringement and non-compliance to data protection laws and regulation. Additionally, one app (Contact Tracer, Russia) uses an insecure implementation of SSL which leads to insecure communication. As indicated, the app could be vulnerable to MITM attacks, which undermines the information security goal of confidentiality and integrity (Jain and Shanbhag 2012).

These vulnerabilities identified within the aforementioned COVID-19 contact tracing apps result in not a robust security performance. While it was difficult to compare the security analysis of COVID-19 contact tracing apps to other generic apps empirically, we compared previous work that analysed the security of android apps using MobSF to determine whether the magnitude of the identified vulnerabilities could be similar to generic apps. Comparing the vulnerabilities detected in work done by (Papageorgiou et al. 2018), it can be noted that COVID-19 contact tracing apps have significant and a high number of vulnerabilities. This can be supported by recent security analysis performed on COVID-19 contact tracing using MobSF by Sun et al. (Sun et al. 2020), which shows the same magnitude of vulnerabilities we identified in our app set. Hence, it can be argued that while other android apps, e.g., m-Health apps contain vulnerabilities, the quick development and release of COVID-19 contact tracing apps without sufficient security analysis tends to result in apps that have a poor security posture.

#### The GDPR impact

As mentioned, our app set contains a variety of COVID-19 contact tracing apps that come from different countries across the globe. Therefore, we aim at interpreting and answering the question of how these apps differ or are similar to each other in terms of their security fulfillment across the EU and rest of the world.

**Fig. 14:**
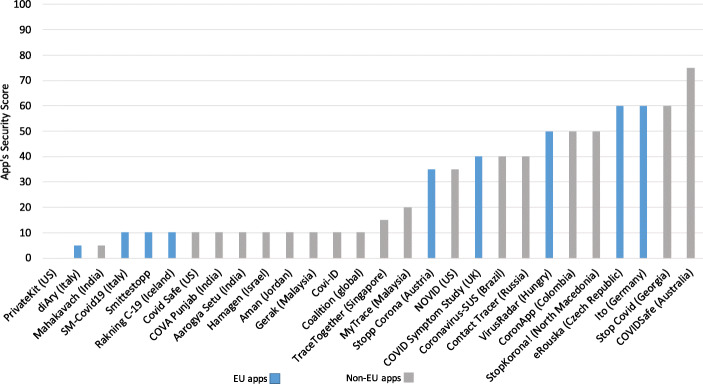
Apps’ security scores: EU vs. Non-EU contact tracing apps (the higher, the better)

Figure 14 shows a comparison graph of COVID-19 apps security score between EU and non-EU apps. The app security – which is as a result of the MobSF measurement – is given a score of 0-100, as manifested in the generated reports; where 0-15 indicates critical risk, 16-40 indicates high risk, 41-70 indicates medium risk, and 71-100 indicates low risk. Overall, it can be noted that majority of the apps have a very poor app security score. However, by taking each area in isolation, several differences can be identified. The EU contact tracing apps show major differences in app security score. While some apps show a medium app security score, for example, eRouska (Czech Republic) with an app security score of 60 and ito (Germany) with an app security score of 60, which is majorly attributed to the fact that they have few vulnerabilities, with even less that have high severity score, four of them show critical security scores. Interestingly, one of these apps with a critical app security score (SM-Covid19, Italy) has also been highlighted as one of the five apps that hungrily accesses dangerous permissions (see Section 4.3.2). These type of permissions go for more sensitive information from the user. Hence, if the vulnerabilities within these apps are exploited, an attacker would be able to access highly sensitive personal data - which would range from location to health status among other personal data.

Despite the fact that these countries are GDPR enforced and some apps tend to access less sensitive resource events, it can be interpreted that the reason for such an app security score is due to the considerable number of vulnerabilities inherent in these apps. This of course leads to several security issues. For example, the static analysis conducted on diAry (Italy) shows that the app contains hard-coded sensitive information and the data stored within the SQLite database is not encrypted; an attacker could gain access to hard-coded credentials thus giving them further access to sensitive data, or they could potentially read sensitive personal information from the unencrypted SQLite database.

When it comes to non-EU contact tracing apps, a majority of them manifest critical app security score, with Private Kit (US) manifesting an app security score of 0. From the report, it is indicated that the Private Kit contains vulnerabilities that have a high severity, for example, the use of a weak hash (MD5), which is mapped in Table 4 under CWE-327, could lead to a breach in confidentiality or privilege escalation when exploited (Jain and Shanbhag 2012). Further, the app uses dangerous permissions for the purposes of contact tracing, which put a user’s security at risk. While this is the case, there are some non-EU apps that perform well in terms of security aspects. Of noteworthy is COVIDSafe (Australia), which manifested a low risk app security score of 75. From the privacy analysis conducted earlier, COVIDSafe shows that it only accesses one dangerous permission. On the other hand, the security analysis shows that it has few vulnerabilities which can be considered as trivial and low risk (apart from the existence of potential hardcoded sensitive information). This indicates that several factors were considered, including the necessary security measures needed for data protection (Cavoukian et al. 2009) prior to its roll out.

## Impact assessment

After conducting a multi-perspective analysis of existing COVID-19 contact tracing apps, we carry out an assessment to quantify and assess privacy and security the impact of the studied contact tracing apps. Such an assessment provides a basis for risk evaluation based on our empirical data and multi-perspective analysis. We calculate the impact as an aggregated score composed of the metrics explained in the previous sections of this article, namely dangerous permission requests (Section 4.1), privacy policy analysis (Section 4.2), dangerous permission usage (Section 4.3), and security vulnerabilities (Section 4.4). We stress that our score is a privacy and security impact indicator, not an assessment of personal data subject impact normally provided with GDPR-demanded data protection impact assessments nor an indicator of how dangerous/friendly an app might be for individuals’ privacy.

### Impact assessment model

We consider a set of *a* apps $\mathcal {A}=\{v_{1}, v_{2}, \ldots , v_{a}\}$ consisting of four subsets *d* ($\mathcal {D}=\{w_{1}, w_{2}, \ldots , w_{d}\}$), *e* ($\mathcal {E}=\{x_{1}, x_{2}, \ldots , x_{e}\}$), *f* ($\mathcal {F}=\{y_{1}, y_{2}, \ldots , y_{f}\}$), and *g* ($\mathcal {G}=\{z_{1}, z_{2}, \ldots , z_{g}\}$), where *w*_*j*_ (1 ≤ *j* ≤ *d*), *x*_*k*_ (1 ≤ *k* ≤ *e*), *y*_*h*_ (1 ≤ *h* ≤ *f*), and *z*_*m*_ (1 ≤ *m* ≤ *g*) show the impact assessment criteria, namely dangerous permission requests, absent clarification of privacy principles in policy texts, idle usage of dangerous permissions, and vulnerability threats.

We also introduce a set of *u* users by $\mathcal {U}=\{d_{1}, d_{2}, \ldots , d_{u}\}$. Let $F=\{f_{v_{1}},f_{v_{2}}, \ldots , f_{v_{i}}\}$ be the set of features for each app *v*_*i*_. Accordingly, $f_{v_{i}}$ consists of ordered quadruples {(*w*_1_,*x*_1_,*y*_1_,*z*_1_),(*w*_2_,*x*_2_,*y*_2_,*z*_2_)…,(*w*_*e*_,*x*_*f*_,*y*_*g*_,*z*_*h*_)}. We determine each feature as an informative element regarding each app. As a result, the set of features related to all apps is defined as $\{ (F_{v_{1}}), (F_{v_{2}}), {\ldots } , (F_{v_{a}})\}$, where $(F_{v_{1}})$ represents the feature $F_{v_{1}}$ associated to app *v*_1_.

We denote the *Impact Value* (*IV* ) regarding each individual feature $f_{v_{i}}$ concerning app *v*_*i*_ (1 ≤ *i* ≤ *p*) while the following condition is met:

1 $$ IV_{v_{i},F_{v_{i}}}=\left\{ \begin{array}{l l } \text{1} & \quad \text{if} \quad F_{v_{i}}=(w_{j}, x_{k}, y_{h}, z_{m}) \\\\ \text{0} & \quad \text{otherwise}, \end{array} \right. $$

where if there is a hit in $F_{v_{i}}=(w_{j}, x_{k}, y_{h}, z_{m})$, then $IV_{v_{i},F_{v_{i}}}=1$, otherwise $IV_{v_{i},F_{v_{i}}}=0$.

To achieve the *Total Impact Score* per app, our goal is to fuse the quadruple impact assessment criteria (see Sections 4.1 to 4.4) as follows:

1. *Dangerous Permission Request Score* (*w*_*j*_): The first impact criteria deals with the impact of dangerous permission requests as discussed in Section 4.1. The *Dangerous Permission Request Score* has an upper limit of 8, since each app can request a maximum number of dangerous permissions as many as 8 permissions detected in our analysis. As it is evident, the higher the *Dangerous Permission Request Score*, the severe the impact for privacy. For the sake of simplicity we have grouped all location- and storage-related permission accesses into LOCATION and STORAGE categories. Further, we have also normalized all the values between 1–100, being 100 means the most severe case (requesting the maximum number of dangerous permissions, i.e. 8 permissions).
2. *Absent Specification in Privacy Policy Text Score* (*x*_*k*_): The second impact criteria deals with the impact of the absence of essential privacy principles in the privacy policy texts of contact tracing apps as discussed in Section 4.2. The *Absent Specification in Privacy Policy Text Score* has an upper limit of 12, since each app needs to cover 12 privacy policy principles. As can be inferred, the higher the *Absent Specification in Privacy Policy Text Score*, the severe the impact for privacy. For the sake of simplicity we have also normalized all the values between 1–100, being 100 means the most severe case (the absence of all privacy principles in privacy policy texts, i.e. the absence of 12 privacy policy principles).
3. *Idle Dangerous Permission Usage Score* (*y*_*h*_): The third impact criteria deals with the impact of idle usage of dangerous permissions at run-time by contact tracing apps as discussed in Section 4.3. Similar to the *Dangerous Permission Request Score*, the *Idle Dangerous Permission Usage Score* has an upper limit of 8, since each app can access a maximum number of dangerous permissions as many as 8 permissions detected in our analysis (the higher the *Idle Dangerous Permission Usage Score*, the severe the impact for privacy). For the sake of simplicity we have grouped all location- and storage-related permission accesses into LOCATION and STORAGE categories. Further, we have also normalized all the values between 1–100, being 100 means the most severe case (accessing the maximum number of dangerous permissions while being idle/running in the background without any user interaction, i.e. 8 permissions).
4. *App’s Security Score* (*z*_*m*_): The fourth (last) impact criteria deals with the impact of detected security vulnerabilities of contact tracing apps as discussed in Section 4.4. The tool used for our analysis generates security scores per app within our data set ranging from 1 to 100, being 100 means the best security performance, however, to make such a score unified and compatible to other scores, we use the inverse percentage, e.g. an app that has a security score of 80 – the more closer to 100 means better security performance – will be inverted to 20 means that the more closer to 0 means better security performance. Therefore, while calculating the *Total Impact Score*, we use the inverted value of *App’s Security Score*, meaning that an app security score of 100 means the most severe case.

We propose the following *Total Impact Score* as the averaged value of all individual $IV_{v_{i},F_{v_{i}}}$ as described above as follows:

2 $$ \mathrm{TotalImpactScore_{v_{i}}} = {\sum}_{i=1}^{n} IV_{v_{i},F_{v_{i}}}/4.  $$

It is worth mentioning that, we presume all the quadruple impact assessment criteria to be equally risky for privacy and security. In addition, we treat the different data sets as contributing equally to the *Total Impact Score* when fusing the results. The reason for giving equal weights to these four criteria is threefold: (1) privacy-by-design (Cavoukian 2010) as a global standard framework demands both strong security and strong privacy that avoids the pretense of dichotomies such as privacy versus security. Hence, our work remains within the intersection of security and privacy aiming at investigating privacy and security quality indicators inspired by legal requirements. That is why our impact assessment scheme is based on the assumption that apps should offer both strong privacy and strong security, and therefore, all these four privacy and security criteria are equal, (2) these contact tracing apps have very clear functionality (breaking the chain of the virus) and for this functionality they must function with the minimum number of dangerous permission requests as their goal should be neither monetizing nor tracking and profiling (as opposed to other apps like social networking ones), and (3) there is a global consensus on how these apps should function under strict data protection goals (as discussed in Section 2.2). As such, we believe prioritizing one or some of these four criteria (which one is is less or more important) will go against the global demand for making these apps function under strict data protection requirements. We would like to mention that the main objective of such an impact assessment scheme is to classify apps according to their privacy and security impact and quantify the impact that each of them might have on individuals’ privacy and security. In fact, the objective of such an impact assessment scheme is not to rank/score apps based on their privacy and security intrusiveness level and to say which one is more privacy-intrusive or more privacy-friendly. Rather, the goal is to see how each app performs when it comes to the impact that has for individuals’ privacy. This is an important point as all these apps are homogeneous in terms of functionality (contact tracing apps) and expected (by law) to function without the need to access sensitive data (e.g. GPS data). As a result, the goal of such an aggregated impact assessment score (combination of four privacy and security criteria) was to see which of these apps show hungriness in requesting and accessing sensitive data (while they are not supposed to be data greedy by law), which ones are more transparent and open to users (while they are required by law to be fully transparent to users), and which ones are more advanced in terms of following security requirements within the app’s code (while they are required to fulfill essential security requirements within the app’s codes).

Based on our analysis, the impact of an app on users’ privacy can be deemed as more severe if it requests higher number of dangerous permissions, has less coverage of essential privacy principle in its privacy policy document, has higher number of dangerous permissions accesses at run-time, and has higher security vulnerabilities. Algorithm 1 details all the aforementioned steps corresponding to the measurement of both individual and total impact scores. 
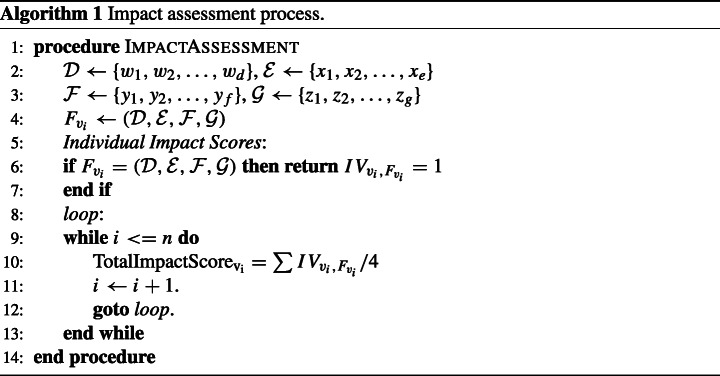


### Impact assessment results

**Fig. 15:**
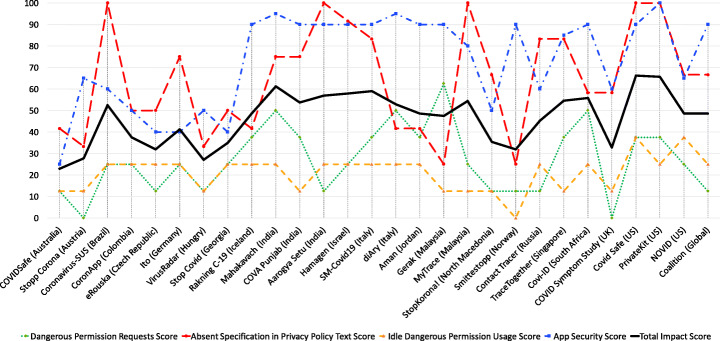
Individual quadruple impact scores (scores are normalized between 0–100): (1) dangerous permission requests score (green), (2) absent specification in privacy policy text score (red), (3) idle dangerous permission usage score (orange), (4) app security score (blue), and (5) the *Total Impact Score* (**black**). The more closer the scores to 100, the more severe the impact on individuals’ privacy

We measured and assessed the scores related to the impact assessment criteria as shown in Fig. 15. We show dangerous permission requests score with green, absent specification in privacy policy text with red, idle dangerous permission usage score with orange, app security score with blue, and the *Total Impact Score* with a thick black line. The more closer the scores to 100, the more severe the the impact on individual’ privacy. As can be seen, the highest scores belong to *Absent Specification in Privacy Policy Text Score* and *App’s Security Score*. This means that these apps have serious issues when it comes to having clear, concise, and regulation-friendly privacy policy texts. Similarly, most apps suffer from critical security issues (as detailed in Section 4.4) that resulted in very high scores (5 apps were scored as maximum, i.e. 100). Although there have been suspicious and abnormal run-time dangerous permission accesses, our results show that apps perform better when it comes to their *Idle Dangerous Permission Usage Score* (the highest and lowest scores are 37,5 and 0, respectively).

**Fig. 16:**
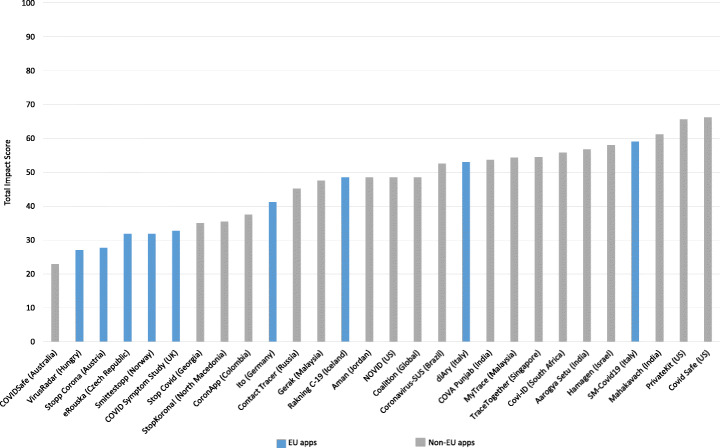
*Total Impact Scores* per contact tracing app (averaged value of individual impact scores which is normalized between 0–100). Ordered from low to high – the more closer the scores to 100, the more severe the impact on individuals’ privacy

As the results from four different sources are aggregated into a *Total Impact Score* as depicted in Fig. 16, an overall comparison can be drawn from it by ordering from lowest to highest impact score. The bar charts are presented for each app. They also differentiate the EU apps from the non-EU ones. An app has the possibility to accumulate a *Total Impact Score* of 100. For instance, the results indicate that Covid Safe (US) has the highest *Total Impact Score* among others (66,2), meaning that it has more severe impact on users’ privacy. The detailed assessment reveals that this app got scores 37,5, 100, 37,5, and 100 (each of them out of 100) for its *w*_*j*_ (*Dangerous Permission Request Score)*, *x*_*k*_ (*Absent Specification in Privacy Policy Text Score*), *y*_*h*_ (*Idle Dangerous Permission Usage Score*), and *z*_*m*_ (*App’s Security Score*), respectively. In terms of apps with less severe impact on users’ privacy, COVIDSafe (Austrlia) has dominated others with a score as much as 22,9 (out of 100). Similar to the observations that we previously had with respect to the better privacy and security behavior of the contact tracing apps published by the EU institutes (where the GDPR is applied and enforced), the results of impact analysis also confirm that 6 (60%) EU apps (shown by blue in Fig. 16) are among the top 10 apps with minimum impact of users’ privacy (only one EU app belongs to the top 10 apps with the most severe impact).

## Discussion

Our multi-perspective privacy and security analysis of COVID-19 contact tracing apps sheds light on a diverse number of data protection issues regarding the existing contact tracing apps. Firstly, we observed that COVID-19 contact tracing apps request a mixed variety of permissions, including dangerous and signature ones. A simple contact tracing app needs to only access a limited number of permissions. For instance, some of the existing frameworks are fully based on BLE technology^12^, and thus, requesting other permissions such as location, microphone, users’ contact details, etc. become unnecessary and irrelevant to deliver the intended functionality – tracing the spread of virus. Surprisingly, we found a great number of apps within our data set that claim they do not require any sensitive permission to function (such as location), but when it comes to their run-time permission accesses, we observed they are actively accessing such permissions. Secondly, the privacy policy text analysis of contact tracing apps showed that these apps tend to be non-transparent regarding their data collection, processing, sharing and transfer practices. This raises serious concerns with respect to their compliance with existing privacy laws such as the GDPR (within the EU) and the corresponding data protection regulation of that country. Thirdly, the run-time analysis of the permission access pattern of these apps revealed that contact tracing apps are massively over-privileged and tend to be data-hungry even though the user was not actively using them. At the first glance, the problem of over-privileged apps is a design issue which is completely independent of national or international data protection legislation. In fact, an app that asks for as many permissions as possible (regardless of whether those permission requests are aligned with its proper functionality) is deviating from basic design principles such as Principle of Least Privilege (PoLP) (Saltzer and Schroeder 1975) that works by allowing only enough access to perform the required job which is also about monitoring and managing access for those who need access such as app developers. Apart from this, the problem of over-privileged apps is directly connected to fundamental legal principles such as data minimization (minimize access to personal data) and purpose limitation (limit access to personal data only for specified purposes) which are widely mandated and enforced by different data protection legislation such as the GDPR, the UK Data Protection Act 2018 (2018), Australia Privacy Act (2020), and Canada Personal Information Protection and Electronic Documents Act (2019). We also believe such a behavior (an app being over-privileged) is an obvious deviation from privacy-by-design (Cavoukian 2010) which is a basic design concept that asks software developers to integrate privacy-respecting measures into the design cycle of their software. Lastly, our study revealed that although there are some apps that behave in a privacy-respecting manner, they suffer from security issues. This is a clear deviation from the *full functionality* principle of privacy-by-design (Cavoukian 2010), that seeks to provide the maximum level of functionality while satisfying all the legitimate objectives in a “win-win” manner by avoiding a false trade-off between privacy and security, indicating the possibility of having both strong privacy and strong security.

Considering the critical situation caused by the COVID-19 pandemic, and the privacy and security risks revealed by our analysis regarding the available contact tracing apps, we would like to highlight that privacy and security considerations should be integrated into the design of contact tracing apps without undermining users’ privacy. Thus, in what follows, we provide several calls for actions regarding existing privacy and security principles enforced by widely adopted data privacy laws such as the GDPR. However, what needs to be highlighted here is that since this study has been conducted in the EU, our work was inspired by the GDPR and other EU regulatory documents. Although there are overlaps between data protection requirements mandated by the GDPR and some other data protection acts enforced in non-EU countries (a particular example of these overlaps is *children protection* which is widely recognized and enforced under different data protection regimes such as US Children’s Online Privacy Protection Rule (1998), Brazilian Data Protection Law (2018), South Korea Personal Information Protection Act (2020), India’s Personal Data Protection Bill (2018), and many more), we would like to highlight that the discussion points discussed within this section may not be necessarily generalizable to all the studied apps (mainly non-EU apps).

### Purpose limitation and data minimization

Our study revealed that COVID-19 contact tracing apps are actively accessing data (including sensitive ones) and sometimes contacting third parties ranging from advertising to analytic networks. For instance, using the tool in (Razaghpanah et al. 2015), we found that Crashlytics (crash reporting and analytics), OneSignal (push notifications), and Facebook Graph (ads and analytics) are the most contacted third-parties by the contact tracing apps (17 apps). Contacting a third-party library by itself is not an obvious sign of a privacy breach, however, our results confirm the high presence of third-party components in contact tracing apps and some of them are associated with third-party advertisement and tracking services (which may to a certain extent justify why many of these apps request and access location-related permissions). We argue that this is not aligned with current EU and non-EU privacy guidelines for designing contact tracing apps. Furthermore, the GDPR states that personal data shall be “collected for specified, explicit and legitimate purposes and not further processed in a manner that is incompatible with those purposes; further processing for archiving purposes in the public interest, scientific or historical research purposes or statistical purposes shall, in accordance with Art. 89 (1), not be considered to be incompatible with the initial purposes”, Art. 5 (1b). When data is collected from users in app ecosystems, such data has to be considered as personal data as in the meaning of the GDPR. The relevant data to the smartphone itself, such as the device’s identifier is also categorized as personal data (ENISA 2017). Therefore, to fully comply with the GDPR Art. 6 (4) (“data processing for incompatible purposes should be avoided unless it is on the basis of a specific set of criteria in the GDPR”), app providers must only process data when the contact tracing app has a specific lawful purpose for doing so. Furthermore, our study shows that there have been many cases where the apps accessed sensitive resources while there was no legitimate reason for that. For instance, those cases that apps are not claiming the need for requesting location data in their privacy policies, while they request and access such permissions in reality is quite problematic, or where they claim they are fully functioning based on BLE technology, but they access location-related permissions (e.g. Coalition). Similarly, even though some of the apps already stated in their privacy policies that they request location permissions (obviously because they are based on GPS technology), we still believe that this is against best data protection practices as our analysis of legal guidelines (see Section 2.2) confirms that active access to location-related permissions is an obvious violation of several data protection principles, including but not limited to data minimization and purpose limitation. More importantly, such an extreme tendency in requesting location-related permissions can result in apps being over-privileged (asking for as much data as they can, even those that are irrelevant to their functionality) and invade individuals’ privacy, such as the claim by a Minnesota law enforcement official confirming that the state was employing contact tracing to identify connections among protesters who were detained during the “Black Lives Matter demonstrations” (2020). Moreover, we observed some of the contact tracing apps request and access signature-level permissions. It should be noted that since signature-level permissions usually give more control to an app through managing system-level functionalities, the developers of contact tracing apps should not request such permission requests unless they are aligned with purposes. We also observed that some of the contact tracing apps do not provide any privacy-by-default options to users to limit/isolate data sharing practices. Hence, data sharing must be isolated by default unless explicitly specified or otherwise chosen by the user.

### Third-country sharing

When sharing data with third-parties, the legal standards of the country where the third-party resides plays an important role. Considering Art. 13 (1f), 14 (1f) of the GDPR, data transfer to other countries is only lawful, where a similar level of protection as provided by the GDPR is guaranteed. Whether this is the case needs to be assessed by the European Commission and can be done on the basis of a country, territory, specified sector, or international organization. Besides these restrictions on where to share personal data, Art. 13 (1f), 14 (1f) require data controllers to adopt appropriate safeguards and means followed by contractual arrangements with the recipient of the personal data approved by the European Commission. In our analysis, we found a number of data transmissions to different countries by contact tracing apps that have not been justified in privacy policy documents. The providers of contact tracing apps need to be fully aware that whenever third-party servers (outside the EU) are used as a back-end, the corresponding regulations applied to third-country data sharing practices are enforced.

### Data retention

Our experiments showed that a significant number of contact tracing apps within our data set (71.4%, 20 apps), are not transparent regarding their data retention period. App providers/developers are required to be clear about the retention period and they should limit it only to the amount of time needed to provide the desired service. Thus, any personal data should be instantly deleted (including stored data on the remote servers) after the expiration of the retention period. Additionally, any confidential data, including location patterns, health-related data, etc. must be successfully deleted upon app deinstallation from the device and any other storage medium.

### Transparency

Transparency is one of the key principles of the GDPR. Smartphone app providers/developers are required to be clear and explicit about their data access, collection, process, and transfer practices. Also, they are responsible for determining the internal rules once data collection purposes change. This also entails the direct communication of such changes to users before they come into effect. Furthermore, any incident regarding users’ personal data shall be promptly communicated to users, e.g., in case personal data breach happens, users and the respective Data Protection Authority must be immediately informed (this also includes the potential occurred risks and possible countermeasures). However, our results confirmed that none of the examined contact tracing apps is transparent about how they react to a data breach, and how they handle such incidents in case users’ personal data falls into the wrong hands. To further improve transparency, a clear, comprehensive, understandable, and legitimate privacy policy text should be accessible to users. We observed some of these apps either do not have any privacy policy document (e.g. Coronavírus - SUS, Brazil) or have very generic texts that do not focus on the app’s data handling practices (e.g. Private Kit, US).

### Integrity and confidentiality

Art. 5 (1f) of the GDPR states that, “personal data shall be processed in a manner that ensures appropriate security, including protection against unauthorized or unlawful processing and against accidental loss, destruction or damage, using appropriate technical or organizational measures (*integrity* and *confidentiality*)”. Developers must make sure that the app’s integrity is preserved intact by checking resources for potential modifications. One way is to restrict writing and modification permissions. However, we found that almost 40% of contact tracing apps tend to not restrict writing and modification capabilities. We observed that nearly 40% percent of the studied contact tracing apps use weak hash functions such as MD5 and SHA1. This is highly critical as it may cause reverse engineering attacks. Additionally, we noticed that 75% of contact tracing apps tend to store raw data on the device’s storage without applying any encryption mechanisms. Enforced by the GDPR Art. 5 (1e), the implementation of appropriate technical and organizational measures to safeguard the rights and freedoms of users is highly important. Hence, app developers have to adopt and apply up-to-date protection and encryption mechanisms for data storing purposes. This is mainly because an insecure storage is not only a risk factor when the device is stolen, but also when another app accesses unencrypted raw data (? VASCO). Therefore, storing any sensitive data such as users’ credentials, location information, etc. on the device’s storage in an unencrypted form must be avoided.

### Accountability

Accountability demands service/app providers to demonstrate how they comply with data protection regulations. This includes careful documentation of all decision-making procedures with respect to the ongoing data processing and conducting Data Protection Impact Assessments (DPIAs) to tackle data protection issues. When personal data is being processed, app developers are required to carefully document all decision making procedures with respect to the ongoing data processing such as maintaining certain documentation on what personal data is processed (how, for how long and for what purpose). A security report handling point (address) must be implemented and maintained aiming at enabling users to contact app developers/providers conveniently. However, our experimental results revealed that 57% of the studied contact tracing apps do not provide any contact information. In addition, all the required procedures must be anticipated and established in case a data breach happens (including a communication channel to react to reports on security and privacy issues). Following best privacy and security practices, all privacy- and security-relevant policies, processes, operations and testing procedures should be documented. This also includes the documentation of risk assessment and management procedures, compliance with regulations and requirements (e.g., the GDPR), a record of users’ consent, objections, contracts with external service providers and third-parties from which the data is collected or transferred to.

We believe the methodology used in this paper can not only provide a quick comparison of COVID-19 contact tracing Android apps’ privacy and security behavior, but also a preliminary assessment of apps’ privacy and security performance in general. This is due to the fact that, the static and dynamic app behavior analysis done in this paper is independent of the nature of the apps. Additionally, the compatibility with privacy regulation is not only meant for COVID-19 contact tracing apps. As such, the data protection principles discussed and covered in this paper are also obligatory for other apps with different functionalities and in different contexts. This also holds for the identified compliance issues.

## Conclusion

Our investigation shows that many of the early contact tracing apps were engineered quickly. They do not take privacy regulation fully into consideration. Their documentations and policies seem incomplete and incohesive. Program code seems poorly quality-assured. We performed various forms of analysis of COVID-19 contact tracing apps based on a group of assessment metrics. Our main findings are:

- Privacy policies provided for many of the apps were found incomplete or non-existent at the time of our study.
- Many apps showed a privacy-invasive permission access behavior, especially concerning location-related permissions.
- Several apps began accessing location permissions even before personalizing and registering them.
- From a regulatory perspective, the EU apps showed in general less privacy risk indicators than the non-EU apps. But still, most apps fail to comply with one or more of GDPR’s privacy principles
- Code vulnerability analysis showed several vulnerabilities in apps’ codes.

The above findings indicate, in our opinion, a very immature state of the infection tracing apps. Incomplete documentation such as privacy policies as well as a large number of vulnerabilities detected by code analysis are strong indicators of quick development. Though speculative, we think this may be the result of a very quick decision-making and of insufficient time for quality assurance. This might also explain that none of the apps covers the complete regulatory requirements set forth by the GDPR - not even the EU apps. Purpose-binding and transparency issues as well as incomplete information for data subject consent have widely been detected in the sample.

### Limitations

Several limitations arise when identifying security and privacy aspects of contract tracing apps. Firstly, our research is limited to the Android platform only. Therefore, it is difficult to speculate about iOS apps. However, we believe the results obtained from privacy policy analysis (see Section 4.2) could be easily generalized to iOS ecosystem as app providers normally publish the same privacy policies for both Android and iOS apps. In terms of resource access pattern analysis, one could think of device instrumentation to jailbreak an iOS device to enable a run-time analysis, but this could then face the incompleteness of analyzed data set (6 out of 28 apps in our data set were not available for iOS). Hence, we abstain from speculating about apps from other platforms. Secondly, we cannot claim that the presented results are reproducible with respect to variable contexts. This is an unavoidable limitation due to the ever-evolving nature of the apps, as well as of the Android platform itself. Apps get regularly updated along with their privacy policies, leaving static data snapshots outdated. Thus, it is a very difficult property to achieve because of the challenges associated with retrieving the older versions of apps, privacy policies, and various forks of the Android operating system to create a similar test bed, as well as identical data collection campaign. However, we have archived the corresponding data that was used to produce results and thus, the analysis could be run again. We also confirm that one should distinguish those apps that were considered to be mature at the time they were deployed and used, and others that were preliminary and not officially endorsed by public authorities, e.g. Ito (Germany). Thirdly, the result from permission analysis suffers from controlled environment that was controlled by avoiding interaction with the device, and therefore, the results of permission access patterns analysis may not be necessarily generalized to other app’s states (e.g. an app being actively used). Lastly, although the results obtained from the vulnerability analysis tool shows a considerable number of security issues, this cannot be fully confirmed as the results may suffer from false-positives. This is due to the fact that, the tool applies regular expression search which might not be accurate enough to figure out more specific and correct security issues.

Furthermore, we encountered several obstacles while preparing the test bed for the apps: *(a)* the official version of the app was restricted to geographic installation only, *(b)* apps were found incompatible to run on our rooted test devices, *(c)* we were compelled to exclude older test devices due to higher requirements (in terms of device and operating system) from the apps, *(d)* apps were found demanding registration with citizen data before running, and *(e)* apps’ documentation was found incomplete, or in languages that are not automatically translatable using regular tools. So, we had to find work-around (e.g. acquiring .apk files from unofficial sources (Apkmirror 2020; Apkpure.com 2020) in order to prepare the test environment. Consequentially, the documented app behavior is expected to deviate from the privacy friendliness/intrusiveness of apps in a real-life device usage scenario. As another limitation of our work, since our study was conducted within the EU, our investigations mostly relied on compliance issues with regard to the GDPR requirements. This means although the results obtained from the legal compliance analysis can be easily generalized to all apps published within the EU, they may not be necessarily generalized to all non-EU countries as those countries may have less strict data protection requirements as offered by the GDPR, and therefore, they may not need to fully comply with all the studied requirements in this paper. In addition, our aggregated impact assessment scheme processes all metrics equally. We believe under certain circumstances some of these metrics may not be fully representative in certain countries where neither the GDPR nor other well-established privacy and security legal requirements are enforced under strict data protection regimes.

### Outlook

A new generation of contact tracing apps is under launch. They are related to a shared code base that focuses on Bluetooth contact tracing with de-centralized local storage on the devices. Cryptography is used to generate pseudonyms, while data subjects are engineered to be in control over data release for tracing in many of the solutions. The apps are launched at the time of the completion of this article. We hope they will be more matured not only in their privacy architecture, but in addition in their documentation and their code quality once we inspect them. We wish their issuing authorities the best with their experimentation with digital contact tracing, which still is an unproven technology.

## Footnotes

1 There is a plenty of COVID-19 related apps published for different purposes ranging from contact tracing to symptom analysis. Throughout this paper, we mainly focus on those apps that are exclusively designed for contact tracing purposes.

2 https://git.cs.kau.se/nurumome/AnalysisOfContactTracingAppsAppendixMaterial

3 ^*3*^ *Full discretionary power (Merriam-Webster dictionary), Accessed 12.06.2020*.

4 https://developer.android.com/reference/android/app/AppOpsManager Accessed 25.09.2020

5 https://developer.android.com/guide/topics/permissions/overview Accessed 28.05.20

6 https://developer.android.com/reference/android/app/ActivityManager.RecentTaskInfo Accessed 12.06.20

7 https://developer.android.com/reference/android/Manifest.permission, Accessed 03.09.2020

8 https://developer.android.com/about/versions/marshmallow/android-6.0-changes.html Accessed 2020-06-11

9 https://developer.android.com/guide/topics/connectivity/bluetooth, Accessed 03.09.2020

10 https://cwe.mitre.org/index.html, Accessed 24.06.2020

11 https://cwe.mitre.org/data/definitions/798.html, Accessed 29.09.2020

12 https://github.com/DP-3T/dp3t-app-android, Accessed 20.06.2020
